# A Systematic Review of Optimization Algorithms for Structural Health Monitoring and Optimal Sensor Placement

**DOI:** 10.3390/s23063293

**Published:** 2023-03-20

**Authors:** Sahar Hassani, Ulrike Dackermann

**Affiliations:** Centre for Infrastructure Engineering and Safety, School of Civil and Environmental Engineering, University of New South Wales, Sydney, NSW 2052, Australia

**Keywords:** optimization algorithms, structural health monitoring, optimal sensor placement

## Abstract

In recent decades, structural health monitoring (SHM) has gained increased importance for ensuring the sustainability and serviceability of large and complex structures. To design an SHM system that delivers optimal monitoring outcomes, engineers must make decisions on numerous system specifications, including the sensor types, numbers, and placements, as well as data transfer, storage, and data analysis techniques. Optimization algorithms are employed to optimize the system settings, such as the sensor configuration, that significantly impact the quality and information density of the captured data and, hence, the system performance. Optimal sensor placement (OSP) is defined as the placement of sensors that results in the least amount of monitoring cost while meeting predefined performance requirements. An optimization algorithm generally finds the “best available” values of an objective function, given a specific input (or domain). Various optimization algorithms, from random search to heuristic algorithms, have been developed by researchers for different SHM purposes, including OSP. This paper comprehensively reviews the most recent optimization algorithms for SHM and OSP. The article focuses on the following: (I) the definition of SHM and all its components, including sensor systems and damage detection methods, (II) the problem formulation of OSP and all current methods, (III) the introduction of optimization algorithms and their types, and (IV) how various existing optimization methodologies can be applied to SHM systems and OSP methods. Our comprehensive comparative review revealed that applying optimization algorithms in SHM systems, including their use for OSP, to derive an optimal solution, has become increasingly common and has resulted in the development of sophisticated methods tailored to SHM. This article also demonstrates that these sophisticated methods, using artificial intelligence (AI), are highly accurate and fast at solving complex problems.

## 1. Introduction

In recent decades, SHM systems have become increasingly popular worldwide due to decaying infrastructure, ever-increasing load demands on existing structures, and the construction of complex new systems. In an SHM system, changes in engineering structures, such as bridges and buildings, are monitored over time using periodic response measurements [1,2,3,4,5]. A typical monitoring system involves two major processes: (I) sensing, to measure structure-dependent data, and (II) data analysis, to identify features from the acquired data allowing the distinction between an undamaged and damaged structure. Ideally, an SHM system fulfills the following requirements: high sensitivity to low damage levels and different types of faults, the capability of continuous monitoring, insensitivity to loading conditions and environmental effects, robustness to measurement of noise, and low installation and maintenance costs. Table 1 outlines the five principal levels of health monitoring, i.e., damage detection, localization, classification, quantification, and prognosis. In general, SHM systems consist of four components: (I) data collection systems, (II) transmission subsystems, (III) information management databases, and (IV) health diagnosis methods. The main two processes in SHM, data sensing and data analysis, are described below:

- Sensing: Sensors are one of the most critical components of any SHM system, and various sensor types are suitable for SHM, such as accelerometers, strain gauges, optical fiber sensors, tiltmeters, and lasers. SHM sensor systems can detect a system’s condition, such as displacement and stress, and assess the effects of environmental variations, including moisture, wind speed, and temperature. The performance of an SHM system depends highly on the data quality measured by the sensor network. Depending on the system requirements, different monitoring strategies are applied, such as strain monitoring, electromechanical impedance monitoring (EMIM), elastic waves monitoring (EWM), vibration-based damage detection, and comparative vacuum monitoring (CVM). Suitable SHM sensors are also used in other fields, such as construction progress monitoring, structural design, safety risk assessment, maintenance management, and smart operations. A comprehensive review of conventional sensor systems can be found in [6], and of advanced sensor systems in [7,8].
- Data analysis: The recorded sensor data typically undergoes a process of data acquisition, signal conditioning, data transfer, data storage, signal processing, and data interpretation. Many data analysis methods have been developed over the years and are constantly being further advanced. The rapid progress in artificial intelligence (AI) and data mining led to a transformation and renewal of data analysis methodologies for SHM. While data analysis techniques, such as traditional signal processing, are applied to datasets to execute and test models and hypotheses, regardless of the amount of available data, AI methods, such as deep learning, uncover hidden patterns in large volumes of data [9]. A comprehensive review of conventional monitoring strategies can be found in [10], and of advanced monitoring techniques in [11].

Research on SHM sensor technologies has been ongoing for decades [12]. Recently, researchers have been increasingly interested in embedding sensors for SHM systems. In [13], Rocha et al. reviewed embedded sensors for SHM systems and discussed sensor characteristics, interactions between sensor materials and host materials, embedding procedures, acquired sensor data, and material behavior. Stoll et al. [14] evaluated the feasibility of embedding eddy current sensors in laser powder bed fusion (LPBF) components for SHM. Environmental and operational conditions (EOCs) pose a significant challenge to SHM systems. For SHM in high-temperature settings, Dutta et al. [15] investigated developments, limitations, applications, and recent advancements in fiber Bragg grating and eddy current sensors. In [16], Simon et al. presented a reliable SHM strategy using radar sensors at 60 GHz embedded in a wind turbine blade and exposed to a defined environment in a climate chamber. Recently, Mieloszyk et al. [17] analyzed the possibility of embedding fiber Bragg grating sensors into additive manufacturing polymeric elements with temperature exposure (both elevated and sub-zero). In recent decades, researchers have discussed different aspects of smart monitoring concepts. Guzman-Acevedo et al. [18] demonstrated the application of global positioning system (GPS) receivers, smartphones, and accelerometers, integrated into a smart sensor system for the SHM of bridges. In [7], Hassani et al. emphasized two main areas relating to smart monitoring: advanced sensing technologies and advanced damage identification algorithms. In addition to highlighting the application of remote and wireless sensing, they showed that advanced data management and analysis techniques are key to managing and interpreting data obtained from monitoring systems.

Characterizing an existing system is only feasible if a minimum amount of data is available. This requirement is directly related to the density of the sensor network, i.e., the least number of sensors that must be placed on the system under evaluation. Sensors must be adequately located, providing optimal data for assessing the structural behavior and status. The measured degrees of freedom (DOF) of complex and large-scale systems must provide sufficient data to characterize the structure’s behavior accurately. Therefore, the following significant challenges must be met in the design of an SHM system: (I) what sensor types to select, (II) how many DOFs to measure, and (III) where to place the sensors. Considering the cost restrictions of an SHM system, decisions have to be made on the sensors’ optimal type, quantity, and locations.

Sensor configuration, also known as sensor placement or deployment, is the fundamental process of constructing sensor networks for monitoring systems. Optimized sensor placement (OSP) improves the overall system performance by enabling optimal system usage, reducing downtime, and avoiding catastrophic failures. The optimization of sensor networks needs to consider multiple aspects to meet the needs of SHM systems. Placing sensors at the most advantageous locations is imperative. Otherwise, incomplete information, such as incomplete modal data, might be recorded, and accurate structural monitoring compromised. The sensor configuration must be specified individually for each system being monitored. Generally, placements can be selected purely based on engineering judgment. However, to support OSP, numerous computational methods have been developed to optimize sensor deployments. Ideally, OSP techniques are able to do the following: (i) reduce the number of sensors, (ii) improve the system’s accuracy, and (iii) provide a robust and optimal design. Comprehensive review articles on the application of sensor placement systems can be found in [19] for aerospace structures, in [20] for the processing industry, and in [21] for the safe operation of nuclear reactors. Many OSP methods have been developed over the years, including effective independence, modal kinetic energy, and information entropy (IE) methods. Detailed descriptions of the most effective OSP methods are presented in the following sections. Although OSP has received some research interest, this only represents 9% of research papers in SHM (according to a keyword search on Google Scholar on 10 March 2023). Consequently, more research is needed to advance OSP techniques that enable accurate, reliable, and cost-effective SHM systems.

Objective functions in OSP problems can be solved using several computational methods, including artificial intelligence (AI) and optimization algorithms. The literature reports many strategies for solving optimal sensor configuration problems, and several approaches have been proposed, including heuristic approaches, intuitive placement strategies, and systematic optimization. More recently, researchers have been combining AI and OSP, known as “Modern OSP”. In [22], Deng et al. proposed an OSP solution based on deep neural network (DNN) for turbulent flow recovery using an ensemble Kalman filter (EnKF). Dinh-Cong et al. [23] developed an OSP reduced order model applying a model reduction technique that employs iterated improved reduced systems (IIRSs). In their research, OSP was applied using the Jaya algorithm. Furthermore, the objective function was created and solved based on a correlation between the flexibility matrix obtained from an original finite element model and its corresponding IIRS matrix.

### 1.1. Background on Optimization Algorithms for SHM

Optimization strategies have existed since the times of Cauchy, Newton, and Lagrange. Newton and Leibniz upgraded differential calculus strategies for system optimization. Bernoulli, Euler, and Lagrange developed the fundamentals and basic concepts of calculus of variations related to the minimization of objective functions. In constrained engineering optimization, unknown multipliers are added to problems. In honor of its inventor, Lagrange, it is named after him. In an unconstrained optimization engineering problem, Cauchy implemented the steepest descent technique. With the development of high-speed digital computers in the late twentieth century, complex optimization processes were implemented, allowing new optimization approaches to be developed. Following these advances, much literature was generated on optimization techniques [24]. In addition, this development led to several well-defined new areas of optimization theory. Optimization algorithms are based on the minimization (or maximization) of an objective function (also referred to as an Error function) E(x). This mathematical function depends on the model’s internal learnable parameters, which provide the basis for calculating target values (Y) based on predictors (X). Different types of objectives can be minimized or maximized, including distance, cost, energy, weight, waste, raw material consumption, processing time, loss, conversion, profit, efficiency, yield, capacity, and utility. An intelligent optimization algorithm with a learning ability is called a learning-based intelligent optimization algorithm (LIOA). An extensive survey of LIOAs was carried out by Li et al. [25], including statistical analysis, classification of LIOA learning methods, and applications of LIOAs in complex optimization scenarios.

The following outlines the typical steps of an optimization process:

1. Definition of the optimization problem.
2. Specification of the objectives to be maximized or minimized.
3. Selection of decision variables.
4. Consideration of restraints.
5. Formulation of final models. Here, the desired goal is defined as the objective function, consisting of variables and constraints, which are functional relations of the inequalities and equalities of the variables.

Optimization methods can be divided into two broad categories: derivative-free optimization (DFO) [26] and derivative algorithms [27].

DFO provides an approach for optimizing over simulations when a closed form of the objective function is unavailable. The theory of DFO algorithms has developed over time, making them useful in a wide variety of practical applications. DFO methods are model-based; they learn the model by evaluating solutions. As a result, the model is used to guide the sampling of solutions in the next round. For example, cross-entropy methods may use Gaussian distributions as models, Bayesian optimization strategies employ Gaussian processes to model joint distributions, and learning algorithms have been incorporated into distribution estimation algorithms.

Derivative-based optimization uses gradient-based optimization techniques to determine the direction of the search based on the derivative information of an objective function. These optimization algorithms can be classified into two main categories:

- First-order optimization algorithms [28]: Optimization algorithms minimize or maximize a loss function (or objective function), E(x), using gradient values. Gradient descent is a widely used first-order optimization algorithm. First-order derivatives can determine whether a function increases or decreases at a particular point. First-order derivatives are lines that are tangential to their error surfaces. First-order optimization techniques are generally time-saving and straightforward calculation methods that converge quickly for large datasets.
- Second-order optimization algorithms [29]: In these algorithms, error functions (or objective functions) E(x) are maximized or minimized using a second-order derivative, also known as the Hessian. The Hessian of a matrix can be considered the partial derivative of the second order of the same matrix. The second order is rarely used, considering the cost of calculating second derivatives. A function’s curvature, also known as the second-order derivative, can be used to determine whether the first derivative is increasing or decreasing. Second-order derivatives are quadratic surfaces where the error surface’s curvature can be touched. In general, second-order optimization techniques are time-consuming and memory-intensive.

For SHM systems, optimization algorithms have been used in many areas, including sensor system design and structural damage detection. As such, optimization algorithms can help to determine the optimal number of sensors and the best sensor locations. A set of objectives is defined, based on the problem’s variables. The error function can be categorized into single- and multi-objective functions. An important issue is the selection of a suitable optimization algorithm, which should be determined based on objective function types. For structural damage detection, objective functions can be used to identify the location and severity of damage in one-step methods or multi-step methods. One-step methods use optimization methods to determine the extent and location of damage in one step, while in multi-step methods the extent and location of damage are detected in more stages.

A summary of review articles on optimization algorithms for SHM systems is presented in Table 2. Since 2000, optimization algorithms have been used in 13% of all publications in the field of SHM (according to a keyword search on Google Scholar on 10 March 2023). A selection of publications on optimization algorithms for OSP and SDD between 2010 and 2023 is provided in two tables. Table 3 presents the use of optimization algorithms for OSP, and Table 4 outlines applications of optimization algorithms for Structural Damage Detection (SSD). Here, various specifications are given, including the investigated optimization algorithm, the analysis type, the damage type, and the monitoring system.

### 1.2. Selection Process and Organization of Papers

**Figure 1:**
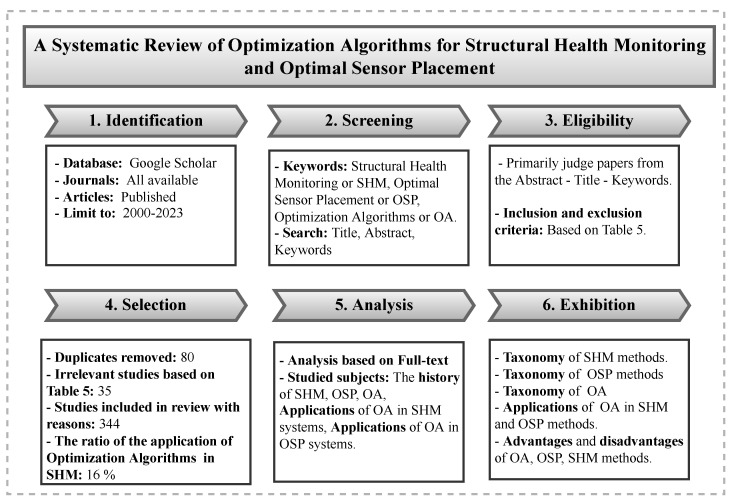
The process for selecting, researching, and analyzing relevant research papers.

**Figure 2:**
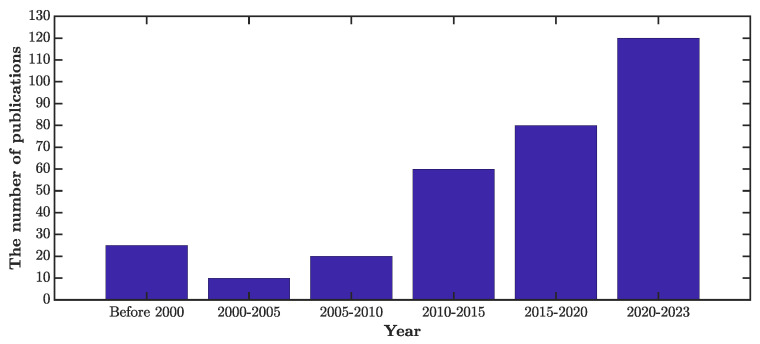
Number of reviewed articles by year.

The selection of relevant papers for a review article is a challenging task. A process flowchart outlining the various steps that guided the article selection process for this literature review is presented in Figure 1. In Table 5, we also provide the inclusion and exclusion criteria in selecting the articles. For this review paper, a total of 344 articles were selected and reviewed. Figure 2 shows the number of reviewed articles subdivided by year. As can be seen, this work focused mainly on recent articles (almost 70% of the reviewed articles were from 2015 to now). Since many journals publish research on optimization algorithms (OAs), SHM systems, and OSP techniques, Table 6 provides researchers with a fast way to find suitable journals related to these subjects. This table gives information about the type of journal (Q1 or Q2) and its founding year. The table also shows the number of articles published so far in each area.

**Figure 3:**
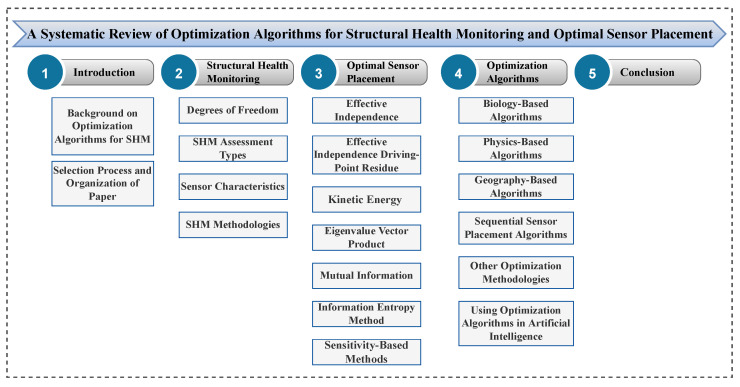
Structure of different sections of the article.

The present article systematically reviews the history of research on optimization algorithm development for SHM systems. The paper’s wealthy literature review investigates numerous approaches and their effectiveness. Moreover, readers are provided with the concept of optimization algorithms and their applications for OSP and SDD methods. Besides all this, as complementary tools, flowcharts, and tables are presented. These summarize the processes of the most important optimization algorithms used for SHM and provide an overview of recent related literature. A diagram of the manuscript’s structure summarizing the different sections of this work is displayed in Figure 3.

## 2. Structural Health Monitoring

In general, an SHM system involves continuously monitoring an engineering structure to identify and analyze changes in the structure’s geometric and material properties over time. In the 1980s, different SHM strategies were developed for various applications, from offshore structures to bridges to aerospace systems. For each application, unique monitoring strategies were developed, focusing on the particular requirements of the structures. Adams et al. [67] published one of the first milestone papers in this field. The authors developed an innovative non-destructive testing (NDT) method based on vibration measurements. Using vibration data from the structure and a suitable theoretical model (acceptance function), it was shown that damage could be identified in a one-dimensional model, estimating its location and magnitude. During this time, Cawley et al. [68] realized that damage causes local stiffness reductions and changes in natural frequencies that can be used to locate the damage. Today’s state-of-the-art in SHM aims to provide accurate and robust systems that can detect, locate, and estimate the size and type of damage and provide remaining lifetime estimations. The health assessment of a system involves identifying four system characteristics: (1) Environmental and operational conditions (EOCs), (2) Mechanical damage or fault, (3) Growth of fault or damage, and (4) System performance after damage or fault.

Recently, Hu et al. [69] proposed a hybrid strategy for damage detection and condition assessment of hollow slab bridge hinge joints using physical models and vision-based measurements. Using this method, the authors determined the damage’s existence, location, and severity based on reduction in the stiffness of hinge joints. In [70], Dessena et al. introduced a novel approach to damage detection and quantification, based on a Kriging approach, to update numerical systems.

The following outlines a general step-by-step technical strategy of an SHM system:

- Step 1: Define the health monitoring problem. This step includes identifying and defining the system requirements, conditions, and limitations, such as the following: loading environment, damage and failure modes, initial damage conditions, system life cycle, warranty and duty-cycle issues, existing sensors, maintenance history, and diagnostic and prognostic requirements.
- Step 2: Develop the SHM models (analytical, numerical, experimental). In this step, SHM models are developed, specifying the following components and transducers: data-driven and model-based approaches, developing failure and damage models, analyzing the sensitivity of components to damage and loads, developing models based on the effects of EOCs and validating and updating models.
- Step 3: Develop and implement measurement systems. This step includes evaluating the information environment (e.g., bandwidth and amplitudes), encompassing the following: defining variables to identify damage and loads, establishing a measurement infrastructure, adjusting actuators and sensors to the optimal positions to determine fault, and calibrating actuators and sensors continuously.
- Step 4: Interrogate information and develop damage identification algorithms. This step includes filtering and processing measurement data, which entails the following: identifying and minimizing sources of computational variability, extracting damage features using models, identifying and reducing variables that are not measured and detecting and quantifying damage and loads.
- Step 5: Develop damage and performance prediction algorithms. This step includes specifying future loading scenarios: selecting damage and failure models, predicting damage initiation and evolution, defining and reducing uncertainty sources in damage prediction and predicting future performance.

**Figure 4:**
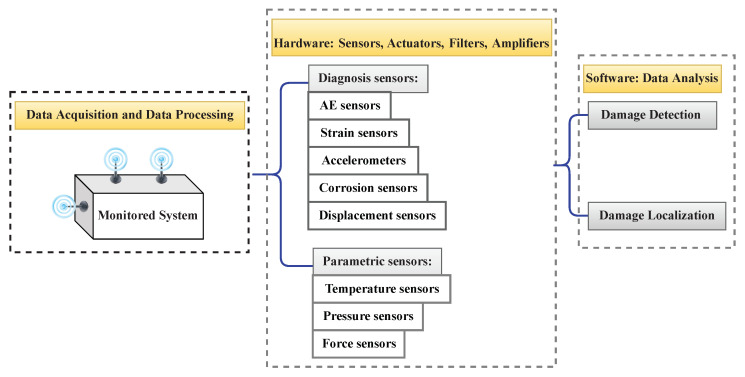
Components of SHM systems.

The two main components of an SHM system are hardware and software. The hardware comprises a dense network of sensors, actuators, filters, amplifiers, and cables. The software components include data processing modules. Figure 4 displays the main components of an SHM system. As a general guideline for developing a suitable SHM system, the following should be considered:

- Types of SHM system
- Types of sensors
- Methods of excitation
- Quantity of sensors and excitation points
- Sensor and excitation locations
- Data transfer type and storage mechanisms
- Types of data acquisition systems
- Information management types
- Types of information interpretation and diagnosis

An SHM method’s success is directly related to selecting these elements. To better understand how these factors contribute to implementing a reliable monitoring system, the readers are referred to [38,71].

Over the years, many SHM strategies have been developed. Today, these can generally be divided into two types of systems: conventional and advanced. Many early studies used conventional methods to identify the extent and location of damage in SHM systems. These methods use vibration tests or numerical simulations to detect damage resulting from changes in the dynamic properties of structures. In contrast, advanced techniques involve more complex methods, such as Hilbert Huang transform (HHT), wavelet analysis, neural networks, and optimization heuristics. Vibration-based damage identification methods are among the earliest proposed SHM techniques widely used for various structures. These techniques are based on the fact that changes in a structure’s mechanical properties reflect changes in dynamic characteristics. Therefore, structural damage, or degradation, affects not only the structure’s mechanical properties, but also changes the dynamic system responses. A typical dynamic property is modal data, which includes frequencies and mode shapes. Using resonance frequencies (natural frequencies) and mode shapes is a popular method for identifying damage (based on dynamic tests). since they are easily obtained and reliable. Hassiotis et al. [72] proposed a technique for identifying localized stiffness reductions in a structure using only natural frequency measurements. An optimization problem, based on eigenvalue sensitivities, was designed to minimize the criteria in regard to changes in the rigidity of elements and residues of eigenvalue problems. Identifying damage in an aluminum beam was possible using only a few natural frequencies.

Hassani et al. [65] developed a new optimization model for damage detection using mode shapes. Their model is based on a sensitivity approach for structures with closely spaced eigenvalues. They demonstrated the superiority of their method, compared to previous studies, in damage detection of complex systems. Fu et al. [73] presented a two-step method to detect local plate damage based on modal strain energy and response sensitivity analysis. By reducing the modulus of elasticity, local damage was simulated. A noise reduction method was proposed in [74], using a variational mode decomposition (VMD) algorithm to reduce white noise interference. In [75], response changes were analyzed using an empirical mode decomposition (EMD) algorithm of the condensed frequency response function (CFRF) contaminated by a high percentage of noise.

Even after about half a century has passed since their initial proposition, vibration-based damage identification methods continue to appeal to researchers, as demonstrated by the works of Liao et al. [76], Wodecki et al. [77], and Machynia et al. [78]. Pothisiri et al. [79] developed a conventional strategy and assessment algorithm based on measured modal response and a finite element model of the system. Kourehli et al. [80] proposed a conventional method for detecting and estimating structural damage based on incomplete modal data and incomplete static responses. A simulated annealing algorithm was used to determine the damage location and severity of the damage in structural elements. Using HHT and an EMD method, Delgadillo et al. [81] presented an advanced bridge damage identification technique, known as “Improvements on Complete Ensemble Empirical Mode Decomposition with Adaptive Noise” (ICEEMDAN). He et al. [82] developed an advanced method that combined echo state networks (ESNs) and multiscale convolutional neural networks (MSCNNs) to extract time–frequency features of civil structures for damage identification. For a comprehensive overview of conventional and advanced methods for SHM systems, we refer the interested reader to [1,83], respectively.

In SHM, Bayesian inference is used for the assessment of structural integrity by updating a probabilistic model based on monitoring data. The authors of Zeng et al. [84] proposed a computationally efficient and likelihood-free Bayesian inference method, BayesFlow, to infer probabilistic damage from SHM models. As part of [85], a sparse Bayesian method of detecting structural damage was proposed that can be applied to standard and nonstandard distributions of probability. The improved Jaya (I-Jaya) algorithm was developed by Ding et al. [86], which incorporated sparse regularization and Bayesian inference into the objective function. In [87], in order to account for measurement noise and modeling errors, a Bayesian system identification framework was used to formulate a likelihood function that connects damage parametric description with scattering estimates to predict scattering properties. For a comprehensive overview of Bayesian inference in SHM, we refer the interested reader to [88].

### 2.1. Degrees of Freedom

As stated above, an SHM system aims to continuously assess a structure to identify structural damage, including material damage and structural stability problems. Structural instability can occur due to joint displacements. The Degrees of Freedom (DOF) of joints are determined by their possible free movements or rotations, i.e., the number of directions in which the joints of a system can cause a displacement. Therefore, DOFs are good candidates for installing sensors to collect data for structural damage detection.

There are two types of DOF in each node of an element: translational and rotational. A system’s component nodes may have rotational and translational degrees. For example, the node of a laminate composite plate has five DOFs, including two degrees of rotation and three degrees of translation [75]. A three-dimensional truss also has three translational DOFs per node [65]. Depending on the system, each node can have different DOFs. Readers interested in DOFs are referred to [89] for more information. The placement of sensors is usually based on the translational degrees, since the measurement of rotational degrees is more challenging and expensive to implement. Due to this, OSP systems are typically aligned with translational degrees.

Numerical experiments generally require three-dimensional finite element models (FEMs) to optimize sensor configurations and to detect damage. Due to the complexity of large structures, the generated FEMs may have thousands of DOFs. Therefore, model simplification and reduction methods are typically used for efficient sensor configuration modeling. For instance, Ni et al. [90] developed an equivalent reduced-order FEM for the Canton tower with 37 beam elements and 185 DOFs. This FEM was also used by Yi et al. [91] for an evolutionary algorithm-based sensor configuration study on the Canton tower.

Many computational applications can generate three-dimensional (3D) FEMs for OSP and SHM systems. FEM is predominantly modeled using ANSYS [91], SAP [92], ETABS [93], ABAQUS [94], Python Chen et al. [95], and MATLAB [75].

### 2.2. SHM Assessment Types

Before choosing the type of SHM system, two main decision factors should be determined: (I) monitoring time frame and (II) monitoring scale. Depending on the time frame, a structure may be under short-term or long-term monitoring. Monitoring scales may also include assessing either (I) a specific problematic location or (II) the entire system. Below we discuss these factors in more detail:

- Monitoring time frames:
  . Long-term monitoring: This monitoring aims to identify structural faults in a system by monitoring its performance over a long period.
  . Short-term monitoring: Assessment methods with a short-term objective, typically involving NDT techniques.
  . Early warning: In this type of monitoring, an early warning alarm is issued when a predetermined threshold is exceeded, informing the user to monitor for possible damage.
  . Inspection: This type of monitoring aims at assessing the condition of a system or its components on a regularly scheduled basis.
  . Collapse warning: This monitoring plan involves the shutdown of the inspected systems when there is a risk of system collapse.
- Monitoring scales:
  . Member monitoring: A specific member of a system is monitored.
  . Local monitoring: A particular region of a system is assessed.
  . Global monitoring: The overall health state of the entire system is monitored.

### 2.3. Sensor Characteristics

Sensors are devices capable of detecting and recording inputs from the physical environment. The input can be heat, light, motion, pressure, moisture, or other environmental phenomena. The sensor output is generally a signal, such as a voltage measurement, that can be converted into a human-readable scale or recorded as a digital quantity, and displayed at the sensor location or sent electronically over a network for further analysis and processing. Specific characteristics of sensors include range, accuracy, sensitivity, stability, static and dynamic specifications, repeatability, compensation of EOC changes, and energy harvesting. An ideal sensor is sensitive to the measured property and insensitive to any other properties they might encounter in their application.

Sensors can be categorized as active or passive. The term “active sensor” refers to one that requires an external power source to react to environmental input and produce output. Weather satellite sensors, for example, require energy to provide meteorological data about the earth’s atmosphere. By contrast, passive sensors do not require an external power source to detect environmental input. Power is provided by the environment itself, such as light or thermal energy. An example of a passive sensor is a mercury-based glass thermometer. Fluctuating temperatures cause mercury to expand and contract, changing the mercury volume in the glass tube. A human-readable gauge is provided outside the glass tube to display the temperature.

A typical health monitoring system is composed of a network of sensors that measure quantities such as stress, strain, vibration, inclination, humidity, and temperature of the structure and its surrounding environment. Over the years, researchers have developed a variety of sensors suitable for SHM based on the latest advances in sensor technology. Generally, sensors in SHM systems fall into two categories: advanced [96] and conventional [97]. The most widely used sensors for SHM include fiber optic sensors (FOSs), accelerometers, vibrating wire traducers, linear variable differential transformers (LVDTs), load cells, strain gauges, inclinometers (slope indicators), tiltmeters, acoustic emission sensors, microelectromechanical systems (MEMSs), and temperature sensors. Table 7 lists the different types of sensors used for SHM systems and the measurement types they perform.

A challenge in designing an effective and efficient SHM system is the choice of suitable sensors, and various factors must be considered. In the following, we provide a list of criteria for the reader to assist in sensor selection.

- System objectives: Any SHM strategy must consider the objectives of the system, such as research, condition assessment, validation of design assumptions, or hazard- specific safety.
- Type of structure: Suitable sensor types depend on specific characteristics of the structure to be monitored, such as the type of materials (e.g., concrete or steel), the design life of the structure, or the location of the structure’s site (e.g., underwater or underground).
- Measurable quantities: The type of data that needs to be measured, i.e., the chemical or physical quantity, further dictates the choice of sensors.
- Sensor specifications: The specifications of individual sensor types are crucial properties to be considered and include sensitivity, resolution, bandwidth, and range of senses.
- Physical sensor characteristics: The accuracy of test results can be affected by physical factors of sensors, including size, weight, strength, and interactions with systems.
- EOCs: Sensors designed for laboratory testing may not be suitable for harsh environments. A sensor must be protected from hostile states when operating in harsh conditions, such as at high or low temperatures, in chloride, in humid conditions, or in acid.
- System cost: The total cost of an SHM system is a crucial factor in designing the sensor network. Costs include costs of sensors, acquisition systems, additional hardware, labor, monitoring duration, system maintenance, and expertise in analyzing information and preparing reports.
- Sensor quantity and placements: To determine the sensor numbers and locations, all of the above criteria must be considered. In addition, it is essential to determine how much redundancy the sensing system should have since sensor failure is unavoidable.

Many sensor types have been investigated and further developed for various applications in SHM systems. Smart wireless sensors (WSSs) have become frontier devices in achieving effective SHM systems, due to their low cost, flexibility, and ease of long-term deployment. The research of Lawal et al. [98] provided a framework for high-fidelity wireless acceleration and strain sensing, commonly known as multimetric sensing. Amaya et al. [99] proposed using embedded FOSs and pattern recognition techniques for SHM in reinforced concrete structures. In a case study presented by Bertulessi et al. [100], an existing water penstock bridge in the Valle d’Aosta Region, Northwestern Italy, was equipped with an SHM hybrid system consisting of Brillouin distributed FOSs (D-FOSs), vibrating wire extensometers, and temperature probes. Aulakh and Bhalla [101] evaluated piezo sensors for operational strain modal analysis. In [102], using smartphones and microscopes, microimage strain sensing sensors (MISSs) were investigated for measuring strain parameters in structural members. Saravanan and Chauhan [103] studied the coupled electromechanical behavior of a smart piezoelectric ceramic Lead Zirconate Titanate (PZT) transducer to determine damage. Using fiber Bragg grating (FBG) sensors in a network, Soman [104] developed a multi-objective optimization technique for actuator and sensor placement. An overview of some recent review papers on sensor systems in SHM systems is presented in Table 8.

### 2.4. SHM Methodologies

**Figure 5:**
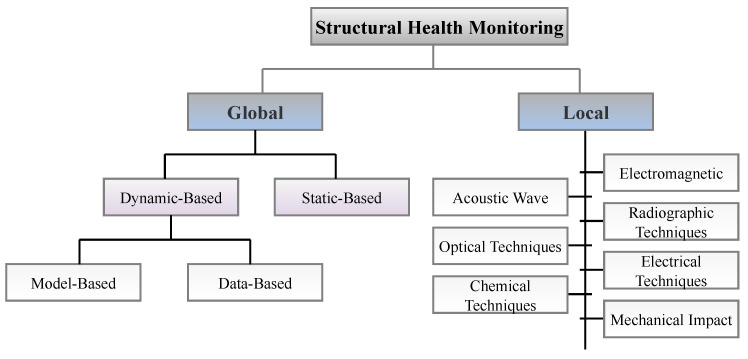
Classification of SHM systems.

SHM methods can be divided into two general categories: global and local. The importance of considering a global monitoring approach is often apparent when a specific structural component cannot be accessed, or the entire structure needs to be evaluated. Figure 5 displays various subdivisions of SHM systems. As can be seen in the figure, global monitoring can be subdivided into static and dynamic categories. Moreover, dynamic-based methods can be further divided into model-based and data-based methods. Data-based methodologies are based solely on measured data from monitoring systems and analytical rules to evaluate the structure and predict how a damage scenario will escalate over time or when a fault will occur. Model-based methods involve solving an inverse problem. Global damage detection methods face two critical challenges: (1) finding a feature that is sensitive enough to detect minor damage and (2) developing methods that are not affected by changes in EOCs.

As a traditional global SHM system, Frigui et al. [111] investigated a new vibration-based damage detection method (VBDDM) to detect and localize damage. This method was tested on a finite element model of an existing building. In [112], a nonlinear vibro-acoustic modulation technique was used for structural damage identification. To illustrate the proposed methodology, nonlinear vibro-acoustic responses from composites were simulated, and data from an impact test was used.

A data-based SHM approach was proposed in [113], by Svendsen et al., for damage detection in steel bridges. In [114], Shi et al. presented a two-dimensional directional continuous wavelet transform (2D-DCWT)-based damage identification algorithm for line-type damage detection in plate structures. Zhang et al. [115] aimed to minimize the influence of modeling uncertainty during model updating so that the updated model could accurately represent damage states. To accomplish this goal, the researchers developed a methodology using pattern recognition techniques to supervise the structural damage identification and guide the Bayesian model updating (BMU). In [116], a new damage detection method for bridges with precast deck panels was developed, based on FE analysis and load testing results. The method successfully identified the location, and significance, of potential deck joint damage by measuring bridge responses and updating models. In a recent study, Ni et al. [117] proposed a likelihood-free Bayesian method for identifying structural parameters. Bayesian inference was performed using a transitional Markov chain Monte Carlo (MCMC) model and an adaptive Gaussian surrogate model (GSM). Many recent review articles, such as [38,118], have provided good insights into global monitoring methods.

For local damage detection, a large variety of NDT techniques can be used, such as the following: visual inspection (VI), infrared testing (IR), acoustic emission testing (AE), electromagnetic testing (ET), liquid penetrant testing (PT), radiographic testing (RT), magnetic particle testing (MPT), ultrasonic testing (UT), film radiography (FR), straight beam ultrasonic testing (SBUT), leak testing (LT), eddy current testing (ECT), magnetic flux leakage (MFL), laser profilometry (LP), alternating current field measurement (ACFM), angle beam (AB), automated ultrasonic backscatter technique (AUBT), holographic testing (HT), laser shearography (LS), computed tomography (CT), digital radiography (DR), computed radiography (CR), electromagnetic acoustic transducer (EMAT), time-of-flight-diffraction (TOFD), long range ultrasonic testing (LRUT), immersion testing (IT), internal rotary inspection system (IRIS), and phased array ultrasonic testing (PAUT). As part of a local monitoring system, Sun et al. [119] presented a hybrid ultrasonic sensing system, named diffuse ultrasonic wave (DUW), to detect damage to railway tracks using a lead–zirconate–titanate (PZT) actuator and an FBG hybrid sensing system. The experimental results showed that DUW signals could detect damage on railway tracks more effectively than the energy-based index. An NDT method, using electromagnetic waves (EMWs), called EMW–NDT, was proposed in [120]. Delamination, cracks, and other defects in CFRP composites were detected using the proposed EMW–NDT method. Su et al. [121] proposed a technique for detecting cavity damage in automated machines using AE tomography, combining the fast-sweeping method with the limited-memory Broyden–Fletcher–Goldfarb–Shanno (L-BFGS) method. Recent review articles, such as [122,123], discuss various local monitoring methods.

Many advanced damage detection techniques are based on artificial intelligence (AI), including machine learning (ML) and deep learning (DL). ML methods can solve two problems: regression and classification. Both problems have been addressed in advanced damage detection approaches. A typical example of AI is big data (BD) management, which uses graphics processing units (GPUs) to interpret and manage large data sets. GPU also provides the possibility of analyzing information using DL methods. Convolutional neural networks (CNNs), long short-term memories (LSTMs), and graph convolutional networks (GCNs) are examples of DL methods used for monitoring systems. Cha et al. [124] proposed a deep architecture of CNNs for a vision-based concrete crack detection method without calculating defect features. Guo et al. [125] presented a DL-based damage detection method for extracting desired features from mode shapes in damaged systems without requiring any hand-engineered features or prior knowledge. Using deep CNNs, Yu et al. [126] proposed a new method based on smart control devices to identify and localize building structural damages. In recent years, DL has been implemented to reduce noise in images and signals. Singh et al. [127] implemented convolutional autoencoders, based on DL, to model noise and denoise ultrasonic images. In their paper, ultrasonic images were quantitatively analyzed using structural similarity index measure (SSIM) and peak-signal-to-noise ratio (PSNR) metrics. The authors in [75,128,129] provide more information on methods for detecting damage based on reducing noise in input signals.

An overview of recent review papers discussing the state-of-the-art in SHM is presented in Table 9. Some recent research articles on new data analysis methods for SHM are listed in Table 10.

## 3. Optimal Sensor Placement

The objective of OSP is to find an optimal subset of measurement locations from a large set of candidates. The resulting sensor layout should be able to accurately represent the system using only a limited number of DOFs, resulting in time and cost savings. The following optimization equation defines the mathematical model of the OSP problem:

(1) $$\begin{array}{c} minf\left(S\right),\;s\in Z^{+}\\ s.t.g(S)=n,\\ S^{lb}⩽S⩽S^{ub} \end{array}$$

where *f* is defined as the error function; *n* is the given limited number of sensors; $S=(s_{1},s_{1},\ldots ,s_{n})$ is denoted as the candidate locations placed at the DOFs of an FE structural model; $Z^{+}$ is the set of positive integers; and $S^{ub}$ and $S^{lb}$ represent the vectors of the upper and lower bound of *S*, respectively.

A typical OSP system can be considered a three-step decision-making strategy:

1. Set sensor quantities: In this step, the number of sensors installed on a system is determined. It defines the most cost-effective method by selecting the optimal number of sensors that still enables accurate system representation.
2. Optimize sensor locations: This step involves deriving the best sensor placements to obtain the most accurate system data.
3. Evaluate sensor layout: In this step, the performance of various sensor configurations are evaluated, based on optimal system representation.

**Figure 6:**
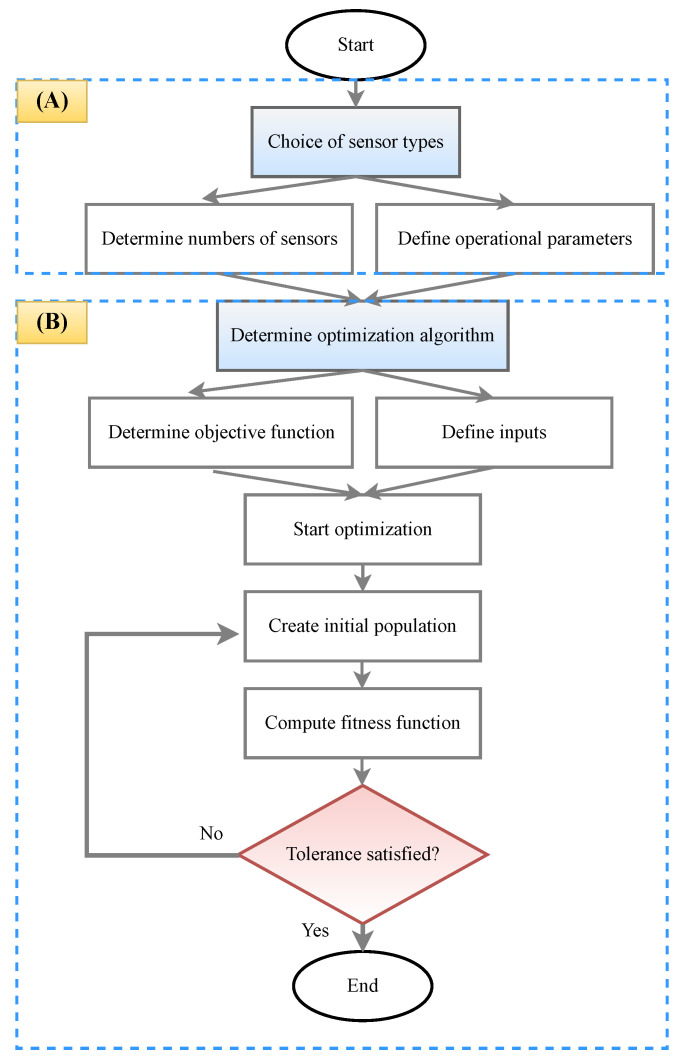
Flowchart for OSP ((**A**): Sensor type and number, (**B**): Defining an optimization algorithm).

The issue of deriving an optimized sensor layout has been studied over many decades, and various strategies have been applied. From the viewpoint of an optimization problem, the sensor locations are the variables in a discrete optimization problem, while the sensor numbers are the constraints. Figure 6 shows a flowchart representing OSP as an optimization problem. Part A specifies the type and number of sensors, and part B defines the optimization algorithm for minimizing or maximizing the objective function.

OSP has a wide range of applications. The concept of OSP was first discussed in electronic science before finding broad applications in structural dynamics and SHM. In [144], Padula et al. provided an overview of OSP for aerospace applications, while Naimimohasses et al. [145] studied OSP for the process industry, and Oh et al. [146] discussed nuclear reactor placement for safety. Sun et al. [147] recently developed a novel discrete optimization scheme based on the artificial bee colony (ABC) algorithm to solve OSP, based on a modal assurance criterion-oriented objective function. A significant challenge of OSP is unmeasured DOFs and harsh environmental conditions. These challenges were recently addressed by Nieminen et al. [148], who proposed a two-phase OSP method that can be applied to commonly used triaxial accelerometers. The proposed method used the minimum variance criterion to estimate structural responses. To minimize the redundancy between the triaxial sensors, the redundancy of information was introduced as an additional criterion for their placement. A proposal for weighting methods, based on modal displacement, was proposed to avoid selecting sensor locations with low vibration energies in an environment with high noise levels. The technique was highly suitable for large-scale FEMs of structures with fine meshes in the industrial sector. In recent years, many review papers have comprehensively addressed OSP systems. For example, Ostachowicz [35] presented an exhaustive review of research studies on sensor placement optimization, comprehensively defining and categorizing the optimization algorithms. Some of the most well-regarded reviews on this topic can be found in [13,106].

**Figure 7:**
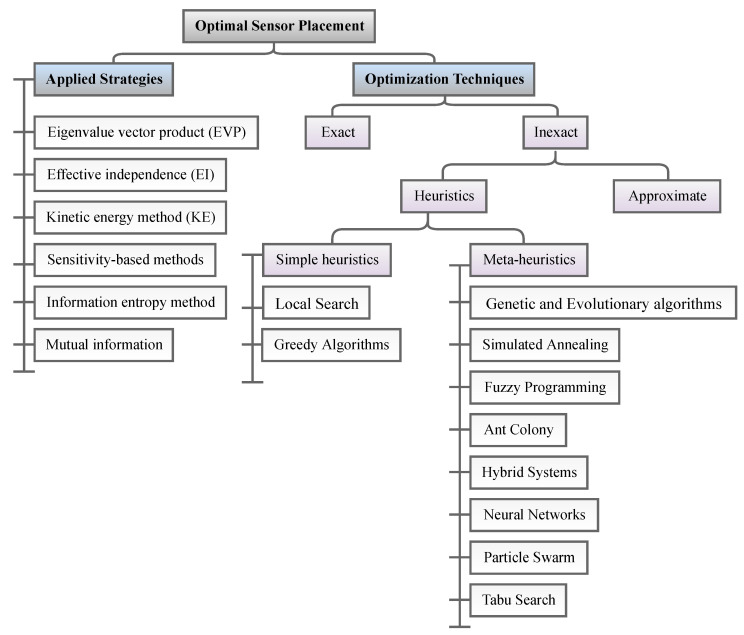
Classification of OSP methods.

A classification overview of different OSP methods is shown in Figure 7, presenting the following methods: effective independence driving-point residue (EI-DPR), effective independence (EI), kinetic energy (KE), Fisher information matrix (FIM), average driving-point residue (ADPR), eigenvalue vector product (EVP), modal assurance criterion (MAC), and strain energy distribution methods. Table 11 reviews recent papers on proposed algorithms used for OSP in SHM. The following sections provide a comprehensive overview of various popular OSP methods.

### 3.1. Effective Independence

The Effective Independence (EI) method is one of the most widely used OSP methods for modal testing and was proposed by Kammer [158]. It is an iterative method based on the Fisher matrix (FIM), providing information about unknown parameters of a sampled random variable. Fisher information refers to the variance of the score regarding the unknown parameter. Multiple unknown parameters can be expressed using these matrices and their elements. Fisher information is defined by:

(2) $${\left(I\left(\theta \right)\right)}_{ij}=E\;\left[\frac{\partial }{\partial \theta_{i}}\;\ln\;f\left(X;\theta \right)\;\frac{\partial }{\partial \theta_{j}}\;\ln\;f\left(X;\theta \right)\right]\;$$

where

- The vector of the unknown is defined by θ = [ $\theta_{1}$, $\theta_{2}$, …, $\theta_{N}$].
- The unknown parameters are $\theta_{j}$ and $\theta_{i}$.
- *X* defines the sampled random variable.
- The likelihood function of θ is defined by $f\left(X;\theta \right)$ = L(θ).
- *E* defines the expectation.

Among a set of candidates, the lowest ranked DOF is eliminated, and the remaining DOFs are ranked based on their contributions to the FIM determinant. The new, reduced, set is iteratively reranked until the desired number of sensors is reached. As a result, this set of optimal placements is accepted. By maintaining the FIM determinant, a collection of linearly independent sensor sites can be selected, retaining sufficient information about target modal responses. This approach is based on the distribution vector $E_{D}$ of EI, which can be represented as the prediction matrix diagonal, *E*:

(3) $$E=\left[\Phi \right]{\left[\Phi \right]}^{T}{\left[\Phi \right]}^{-1}{\left[\Phi \right]}^{T}$$

While the matrix of FE target modes is defined as Φ, it can be partitioned using sensor distribution. A diagonal element determines each sensor location’s fractional contribution to rank *E*, which can be the full rank when the target mode partitions are linearly independent. As an iterative approach, the terms $E_{D}$ are sorted according to the least important sensor, removing the least important ones each time. The corresponding matrix elements and relevant features are also removed. The iterative algorithm continues until the required number of sensors is reached.

Yang et al. [159] presented an interval-EI technique for OSP with uncertain structural information. The paper treated uncertainties as non-probability intervals to overcome the insufficient statistical description of uncertain parameters. The study considered eliminating steps with uncertain cases using the iterative process of the classical EI method. So, their FIM method was extended to interval numbers, which would be more compatible with engineering. Li et al. [160] addressed the inherent relationship and comparison between KE and EI, which are two influencing methods in OSP. KE’s connection to the EI method was studied by analyzing OSP with the EI method from the perspective of a new reduced system. The derived relationship was then verified by applying both methods to the I-40 Bridge, located over the Rio Grande in Albuquerque, New Mexico. Comprehensive reviews have been published providing more detailed information on the EI method [160,161].

### 3.2. Effective Independence Driving-Point Residue (EI–DPR)

The greatest weakness of the EI method is that it is vulnerable to high noise conditions, since the algorithm can only select sensor locations with low signal strength. Average driving-point residue (ADPR) can provide a contribution measure of each point to the overall modal signal.

In this case, if *j* = 1, …, *N* interest modes should be measured, and $\omega_{j}$ is the *j*th mode eigenvalue, ADPR in the *i*th DOF can be calculated as follows:

(4) $${ADPR}_{i}=\sum_{i=j}^{N}\;\frac{\Phi_{ij}^{2}}{\omega_{j}}$$

The EI–DPR vector can be obtained by weighting the EI algorithm values with ADPR values. Considering the *i*th DOF:

(5) $$E_{D_{i}}^{\mathrm{EI}-\mathrm{DPR}}=E_{D_{i}}^{\mathrm{EI}}\;{ADPR}_{i}$$

Chang et al. [162] proposed a methodology to derive the optimum number and location of sensors for bridge SHM and compared their method to other OSP techniques. The results showed that the EI–DPR method concentrated sensors at the midspan, while the KE and EI methods located sensors uniformly throughout the investigated structures. Three examples were used to verify the proposed framework: (1) numerical simulations of a supported beam, (2) FEMs of the Northampton Street Bridge (NSB), and (3) wireless sensor data from the Golden Gate Bridge (GGB). More information about the EI-DPR method can be found in several comprehensive reviews [36,163].

### 3.3. Kinetic Energy (KE)

The KE approach is based on the principle that if any sensor is placed at a point of maximum KE, the sensor has maximum ability to measure the modes of interest. Possible sensor locations are ranked, based on their dynamic contribution to the target mode shapes. There is a critical difference between this method and the EI method, i.e., instead of analyzing the FIM determinant, a KE measure is maximized here. Engineering literature refers to this method as the kinetic energy method (KEM) or modal kinetic energy (MKE). For all candidate sensor sites, KE indices can be determined as follows:

(6) $${KE}_{ij}=\Phi_{ij}\;\sum_{s}\;M_{is}\;\Phi_{is}\;\omega_{j}^{2}$$

with *M* representing the mass matrix. Sensor locations with the highest KE index are selected. The signal-to-noise ratio of this method is higher than that of the EI method, because it chooses sensor locations with the largest available signal amplitudes. Since this method can handle high noise levels, it has been widely applied to real-world structures in noisy environments. However, unlike the EI method, the KE method does not consider the linear independence of the target modes, which is an essential consideration for test–analysis correlation and modal identification.

The number and location of measurements in experimental modal testing greatly influence the quality of the results. Thus, Heo et al. [164] proposed an improved KE optimization method and applied the technique to experimental data derived from an asymmetric long-span bridge model. A comparison was made between the algorithm proposed in this paper and the EI algorithm using experimental data from the bridge model. A detailed description of the KE method can be found in [36].

### 3.4. Eigenvalue Vector Product (EVP)

The EVP method calculates the product of eigenvector elements to derive sensor locations from *N* modes. The best sensor locations are those with the highest values of EVP. An advantage of EVP is that it avoids locating sensors on node points of a mode, maximizing the vibration energy of the resulting sensor layout. The EVP of the *i*th DOF can be calculated as follows:

(7) $${EVP}_{i}=\prod_{j=1}^{N}\;\left|\Phi_{ij}\right|$$

Yang et al. [165] proposed a novel non-probabilistic sensor placement method for SHM, combining the EVP and EI methods. To optimize sensor positioning, the researchers proposed to use interval numbers based on the Relationship of Interval for Sensor Number (RISEN) index and an algorithm based on iterative multi-objective optimization. Four examples were studied, validating the method. Tan and Zhang [36] presented a comprehensive review of the EVP method, providing more details.

### 3.5. Mutual Information

This method measures how much data is learned from one sensor location to another by using mutual information. In its definition, *A* and *B* are two measurement sites, which refer to the amount of information $a_{i}$ retained about $b_{j}$ while considering $I(a_{i},b_{j})$.

(8) $$I\left(a_{i},b_{j}\right)=\log_{2}\left[\;\frac{P_{AB}\left(a_{i},b_{j}\right)}{P_{A}\left(a_{i}\right)\;P_{B}\left(b_{j}\right)}\;\right]$$

where

- $a_{i}$ and $b_{j}$ are the measurements from locations *A* and *B*, respectively.
- $P_{A}\left(a_{i}\right)$ and $P_{B}\left(b_{j}\right)$ are the individual probability densities for data *A* and *B*, respectively.
- The joint probability density for data *A* and *B* is defined by $P_{AB}\left(a_{i},b_{j}\right)$.

$I(a_{i},b_{j})$ becomes zero only if the data of $a_{i}$ is thoroughly independent of the data of $b_{j}$. By averaging all sensor sites, the average of mutual information between *A* and *B* can be calculated, and by minimizing the mutual information between recorders, the results of OSP can be reached. Gierlichs et al. [166] provide more details of the mutual information analysis.

### 3.6. Information Entropy Method

The best location for sensors is obtained by minimizing the variation in information entropy H(D), which is determined by the following equation:

(9) $$H\left(D\right)=E_{\theta }\left[-\ln\;p(\theta |D)\right]=-\int p\left(\theta |D\right)\;\ln\;p\left(\theta |D\right)\;d\theta$$

where

- θ is the uncertain parameter set (e.g., stiffness parameters, modal parameters, etc.)
- *D* is defined as the information of the dynamic test.
- $E_{\theta }$ is the mathematical expectation concerning θ.

Information theory minimizes uncertainty in model estimates. The information entropy method puts more significance on the information content, allowing exact quantification of energy. This is considered a crucial distinction from the EI method, as, in this method, the chosen DOF is given relative importance, while the information entropy is related to the total maximum limit of the entropy. Said et al. [167] presented a novel metric based on information entropy for optimizing sensor placements to detect impact in a composite plate, and used GA to optimize impact detection using strain measurements. Ye et al. [168] proposed an information entropy-based sensor placement method for damage detection and tested it on the Canton tower and the benchmark model. Pei et al. [169] proposed a conditional information entropy-based OSP technique to separately investigate the influences of noise in measurements and the model error in multi-dimensional sensor placement. Golan and Maasoumi [170] provide more detailed information on the information entropy method.

### 3.7. Sensitivity-Based Methods

In general, sensitivity-based methods analyze how different sources of uncertainty in a mathematical model contribute to the model’s overall uncertainty. As an extension of the EI strategy, the prediction *E* matrix is adjusted to apply a sensitivity matrix created for the location of damage. Below is a formula for calculating the modified *E* matrix:

(10) $$E=\left[F\left(K\right)\right]{\left[F\left(K\right)\right]}^{T}{\left[F\left(K\right)\right]}^{-1}{\left[F\left(K\right)\right]}^{T}$$

F(K) is defined as a sensitivity coefficient vector of mode shape variations corresponding to damage vectors. Following the explanation of the EI approach given earlier, the E diagonal terms represent the fractional contribution of the corresponding record site to the E rank. The location with the least contribution is deleted, and this process is continued as an iterative algorithm until the required number of sensors remains.

Based on sensitivity-based methods, Sun et al. [171] developed a novel OSP to identify the number and location of three types of sensors: accelerometers and Fiber Brag Gauges (FBGs), which are commonly used in vibration tests, and piezoelectric sensors (PZT), which are commonly used in SHM using active sensing. Liu et al. [172] proposed a novel sensitivity-based method for determining the minimum number and optimal locations of sensors. The local sensitivity matrix of the recorded outputs to initial states was applied as a measure of observability. The minimum number of sensors was determined, based on the full-column rank of the local sensitivity matrix. The subset of sensors that satisfied the full-rank condition and provided the maximum degree of observability was considered OSP. More details on sensitivity-based methods are provided in [36].

## 4. Optimization Algorithms

As introduced in Section 1.1, optimization procedures can be employed to optimize processes in many different disciplines, including civil, mechanical, power, medical, chemical, electrical, electronics, and industrial engineering. Optimization Algorithms (OAs) help reduce the cost and risk of engineering design and operation and are applied to the design of multi-phase reactors, flow systems, SHM systems, neural networks, sensor detection systems, image processing, or manufacturing processes. The concept of optimization can be explained in several ways:

- Optimization describes and predicts the behavior of a process and is implemented with a mathematical model.
- Optimization aims to find decision variables that minimize or maximize one or more objectives while satisfying constraints.
- The choice of the optimization technique and the formulation of the objective functions affect the reliability of optimal solutions.
- Optimization can effectively estimate unknown parameters, especially in complex nonlinear processes.

An optimization problem consists of three major components: the objective function, variations, and constraints.

- Objective function: In an optimization problem, the objective function (or error function) is iteratively minimized or maximized. The objective function is a linear or nonlinear equation that can also be a single numerical quantity. The objective can be various issues, such as the effective return on a stock portfolio, the time of vehicle arrivals at a specified destination, profits or costs of a company’s production, or the vote share of a political candidate.
- Variations: The quantities or variables that optimize the error function are termed variations. They include various parameters, such as the amount of stock to be bought or sold, the advocated policies by a candidate, or the route followed by a vehicle through a traffic network.
- Constraints: The optimization problem constraints limit its variables (limits of variables). A simple example of a constraint in a production process is that it cannot use less than zero resources and cannot use more resources than are available.

**Figure 8:**
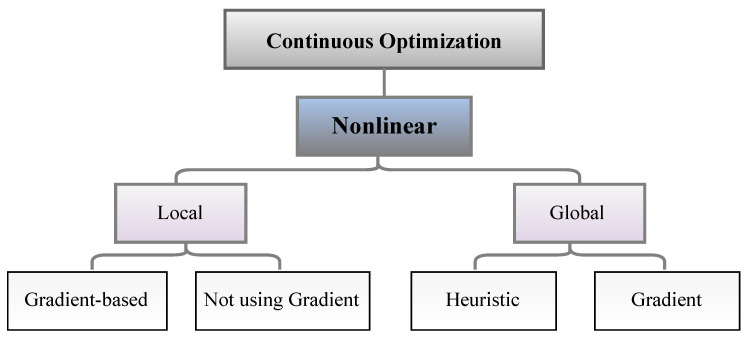
Classification of global and local optimization algorithms.

Optimization algorithms can be classified based on different factors, such as global and local, nondeterministic and deterministic, unconstrained and constrained, one-dimensional and multidimensional, and nonlinear and linear. As shown in Figure 8, algorithms are commonly classified as global and local. Table 12 summarizes the advantages and disadvantages of global and local OAs.

Over the years, many optimization techniques have been developed, and based on their complexity and efficiency, they can be divided into two main categories:

- Traditional optimization techniques [177]: These algorithms are deterministic algorithms that follow specific rules to move from one solution to another. Many engineering design problems have been successfully solved using these types of optimization, such as the following: geometric programming, dynamic programming, nonlinear programming, generalized reduced gradient method, and quadratic programming, etc. The two general divisions of these methods are as follows:
  . Derivative algorithms: hill-climbing algorithms, including gradient descent and Newton’s method.
  . Derivative-free algorithms: trust-region or pattern search methods.
  Despite the widespread use of traditional optimization methods for mechanical design optimization, these techniques are ineffective across a broad spectrum of problems. This is primarily due to their tendency to find local optimal solutions, which are not suitable for solving multivariate problems.
- Advanced optimization techniques [178]: These algorithms are based on stochastic approaches with probabilistic transition rules. Implementing these methods is relatively new and gaining popularity, since they offer properties that deterministic algorithms do not have. These algorithms are also known as metaheuristic algorithms and include the following: differential evolution (DE), evolutionary algorithm (EA), harmony elements algorithm (HEA), genetic algorithm (GA), Hybrid (Hy) algorithm, biogeography-based optimization (BBO), particle swarm optimization (PSO), swarm intelligence (SI) algorithm, artificial immune algorithm (AIA), artificial bee colony (ABC), simulated annealing (SA), differential evolution (DE), harmony search (HS), cuckoo search (CS), and firefly algorithm (FA), artificial bee colony (ABC), Tabu search (TS) algorithm, genetic programming (GP), monkey algorithm (MA), cooperative–competitive evolutionary algorithm (CoEa), expectation-propagation (EP) algorithm, firefly algorithm (FA), whale optimization algorithm (WOA), mixed-integer linear programming optimization (MILP), hybrid metaheuristic optimization algorithm (HGACS), modified TLBO algorithm (MTLBO), and multi-objective evolutionary algorithm (MOEA).

**Figure 9:**
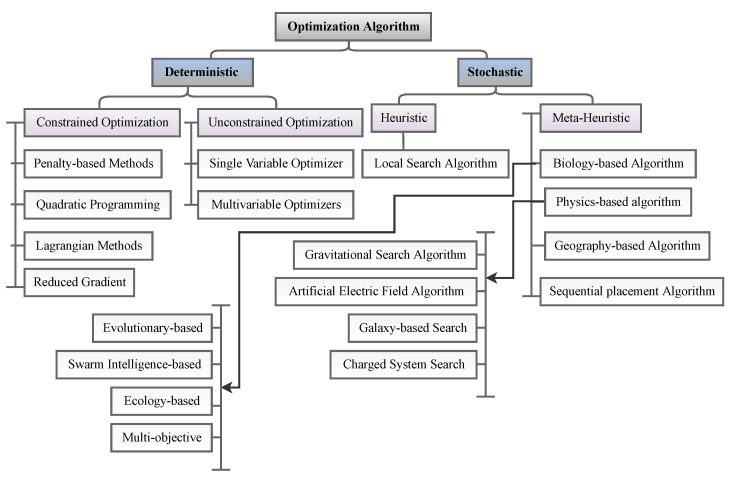
Classification of optimization algorithms.

In SHM, optimization algorithms can solve different objective functions and be used for OSP and at each of the four damage identification levels of a structure, i.e., age detection, localization, quantification, and even lifetime prediction. Figure 9 provides a classification overview of the most recent optimization algorithms. To assist in choosing the latest and most suitable optimization algorithm for the design of SHM systems, we summarized the advantaged and disadvantages of each of these methods in Table 13 and compiled a table (Table 14) presenting all optimization algorithms sorted by their year of development. Optimization methodologies used for SHM systems were reviewed by Gomez et al. in [11,179].

Due to the plethora of available optimization algorithms, selecting the most suitable algorithm can be challenging. Several criteria need to be considered in the algorithm selection process, including feature robustness, probability of finding the global optimum, ease of setup, number of function evaluations (speed), and accuracy of the answer. Many researchers developed methods and guidelines for choosing the best optimization method. For example, Rice formalized the algorithm selection problem in [180], where selection mapping is learned from a set of problems containing certain features. The most appropriate algorithm is subsequently selected from the available set of algorithms.

In general, algorithms can be selected either based on theoretical or empirical considerations. In the theoretical algorithm comparison, only one function class is considered [181]. In the more common empirical algorithm selection, algorithms are ranked according to their performance, and an automated recommendation of the most appropriate algorithm is based on, for example, a specific function [182] or a set of functions, so-called benchmarks. In regard to benchmark selection, a wealth of literature has been compiled [183], ranging from benchmarks based on mathematical functions to benchmarks based on real-world instances. Nevertheless, the results obtained from a particular benchmark cannot be easily generalized to other problems not included in the benchmark.

Numerous comparisons of optimization algorithms have been conducted [184,185]. However, comparing all optimizations correctly and fairly is impossible, since each optimization algorithm is optimized for a particular objective function, i.e., individual optimizations are tailored to specific problems. An algorithm may perform excellently in one kind of problem and perform poorly in another kind of problem. Therefore, the type of objective function is the essential criterion for choosing an optimization algorithm. An overview of articles comparing optimization algorithms is presented in Table 15.

**Table 13:** Advantages, and different types, of optimization algorithms.

Type	Refs.	Advantages	Disadvantages
GA	[186,187]	- Convergence with low probability to local maxima or minima; - Insensitive to target functions of a specific type; - Possibility of parallel and distributed implementations; - Sensitive to parameters in a string of bits, not values; - Relies on probabilistic transition rules; - Uses objective function information, not derivatives.	- Is complex, especially in multi-objective optimization issues; - Long computation time; - Premature convergence may occur due to fitness function coding.
PSO	[188,189]	- Fast convergence, especially in improved PSO (IPSO) models; - Simple implementation and supported platforms; - Time efficient compared to GA; - Practicality in solving multimodal and nonlinear functions; - Improved versions can solve high-dimensional problems.	- Effects of high inertial weight on optimal convergence; - Possibility of convergence to a local optimum, especially at large inputs; - Cannot optimize discrete problems; - Inferior to GA in terms of commercialization and maturity.
SA	[190,191]	- Applicable for large, complex, and highly nonlinear optimization; - Has flexibility and guarantees optimal global convergence; - Known as a versatile programming, and complete, algorithm.	- Inverse relationship between computational time and solution quality; - Not efficient in smooth and minor optimization problems; - Sensitive to the rate of initial temperature change in its initializations; - High computational cost, especially for large data sets.
MILP	[192,193]	- Simple implementation and supported platforms; - High rate of convergence and low gap percentage compared to heuristic optimization methods; - Guaranteed global optimal convergence; - Ability to formulate, especially for different constraints.	- Sensitive to nonlinear system effects; - Low-quality solutions; - No balance between computing time and accuracy; - Sensitive to a large number of binary variables.
DE	[194,195]	- Better performance compared to GA; - Uses a combination of the same population chromosome in the formation of a new generation.	- Hardly any chromosomes of the previous generation are carried forward to the next generation. However, better results can be achieved. - Mutation and crossover operations are performed in one process.
ABC	[196,197]	- Self-organizing; - Collective intelligent data; - Few control parameters; - Fast convergence; - Employs both exploration and exploitation	- Search space limited by initial solution (normal distribution sample should be used in initialization step); - Abandons poor solutions; - Poor local search ability.
ACO	[198,199]	- Simple implementation; - Derivative free; - Good global convergence properties; - Stable optimal result.	- Uncertain convergence time; - Low computational performance and accuracy of the original ACO.
MA	[200,201]	- Able to search globally; - Able to efficiently search locally; - Generates optimal solutions with a higher level of stability.	- Originally designed for problems with continuous variables.
TS	[202,203]	- Quick convergence; - Flexible algorithm; - Good-quality solutions are provided by the algorithm; - Secondary designs are provided by the algorithm.	- Some algorithm parameters need adjustments to find a good solution; - Penalty parameters must be used to satisfy the mathematical model’s constraints; - Re-running the algorithm could change the obtained design.

**Table 14:** Development year of optimization algorithms.

1950–1990	1990–2000	2000–2005	2005–2010	2010–2015	2015–2023
**Evolutionary Algorithms**					
GA; SA; TS	GP; ES; MA; CA; DE; EP; CoEa	GEA	ICA	TLBO; FPA;	SCA; MCEO; ASA; GSO
**Swarm Intelligence Algorithms**					
	PSO	AFSA; HBO; TCO	ACO; SFL; MS; DPO; FA; BA	FFO; KH; CS; BMO; GWO; SLCA; ALO; DA; MFO	IAPSO; WOA; SSA; GOA; HHO; FSO; BWO; CSO; HOA; ISSA; FSA
**Hybrid Algorithms**					
					CBO-PSO; PSO-CS; GWO-SCA; PSO-GWO; MFO-GSA; PSO-WOA; WOA-SA; SA-MFO; SCA-TLBO; HDPSO; PSO-SCA

**Table 15:** Comparison of optimization algorithms.

Ref.	Year	Optimization Algorithms	Description
Wetter and Wright [204]	2004	Discrete Armijo gradient algorithm, GA, PSO, and Hooke–Jeeves algorithm,	Based on the results of this study, it was revealed that the biggest cost reduction is achieved by combining particle swarms and Hooke–Jeeves algorithms. Additionally, it was shown that a simple GA is an excellent choice if a user is willing to accept a slight reduction in accuracy for the benefit of fewer simulations.
Hassan et al. [205]	2005	PSO and GA	The results revealed that both PSO and GA produced high-quality solutions, with quality indices of more than 99% confidence levels. However, the computational effort required to reach such high-quality solutions by PSO was lower than the computational effort required by GA.
Bandyopadhyay et al. [206]	2008	AMOSA, NSGA-II, and PAES	In the study, SA-based multi-objective optimization algorithm (AMOSA) performed better in most cases than MOSA or non-dominated sorting GA II (NSGA-II), while Pareto archived evolution strategy (PAES) performed poorly in most cases. In complex cases, AMOSA was less time-consuming than NSGA-II. Further, AMOSA performed much better than NSGA-II regarding problems with multiple objectives.
Yildiz [207]	2013	GA, PSO, Immune algorithm, HTDEA, ABC, and DE algorithm	According to computational results and discussions, the hybrid technique based on DE algorithm (HTDEA) was an effective optimization method for solving structural design problems more efficiently than other algorithms.
Civicioglu and Besdok [208]	2013	CK, PSO, DE and ABC algorithm	Comparing the CK algorithm with the DE algorithm revealed that the CK algorithm was very successful at solving problems. The DE algorithm acquired a global minimizer with lower run-time complexity and needed fewer function evaluations than the comparison algorithms. PSO and Cuckoo-search (CK) algorithms were statistically more similar to DE than ABC algorithms in performance. CK and DE provided more reliable and precise results than PSO and ABC algorithms.
Hamdy et al. [209]	2016	pNSGA-II, MOPSO, PRGA, ENSES, evMOGA, spMODE-II, and MODA	In the study, the pNSGA-II, MOPSO, Two-phase optimization using the GA (PRGA), Elitist non-dominated sorting evolution strategy (ENSES), Multi-objective evolutionary algorithm, based on the concept of epsilon dominance (evMOGA), Multi-objective DE algorithm (spMODE-II) and Multi-objective dragonfly algorithm (MODA) were run 20 times with a gradually increasing number of evaluations, indicating that the PRGA algorithm explored a large part of the solution space and quickly produced close-to-optimal solutions with good diversity.
Dogo et al. [210]	2018	SGD, vSGD, SGDm, SGDm+n, RMSProp, Adam, AdaGrad, AdaDelta, Adamax and Nadam	The results showed that Nadam performed best across all three datasets, whereas AdaDelta performed worst, compared with Stochastic Gradient Descent (SGD), Root Mean Square Propagation (RMSProp), Adaptive Moment Estimation (Adam), Adaptive Gradient (AdaGrad), Adaptive Delta (AdaDelta), Adaptive moment estimation Extension based on infinity norm (Adamax) and Nesterov-accelerated Adaptive Moment Estimation (Nadam) optimization techniques
Zaman and Gharehchopogh [211]	2022	PSO and PSOBSA	It was shown that IPSO with the backtracking search optimization algorithm (PSOBSA) performed better than other well-known metaheuristic algorithms and PSO variants on almost all of the benchmark problems in terms of global exploration ability and accuracy.
Tawhid and Ibrahim [212]	2023	CS, MBO, and MBOCS algorithm	The study showed that the hybrid swarm intelligence optimization (MBOCS) algorithm could overcome the disadvantages of monarch butterfly optimization (MBO) and CS algorithms. Compared with other algorithms, the MBOCS algorithm outperformed the others and was a competitive and promising technique for solving complex optimization problems.

**Figure 10:**
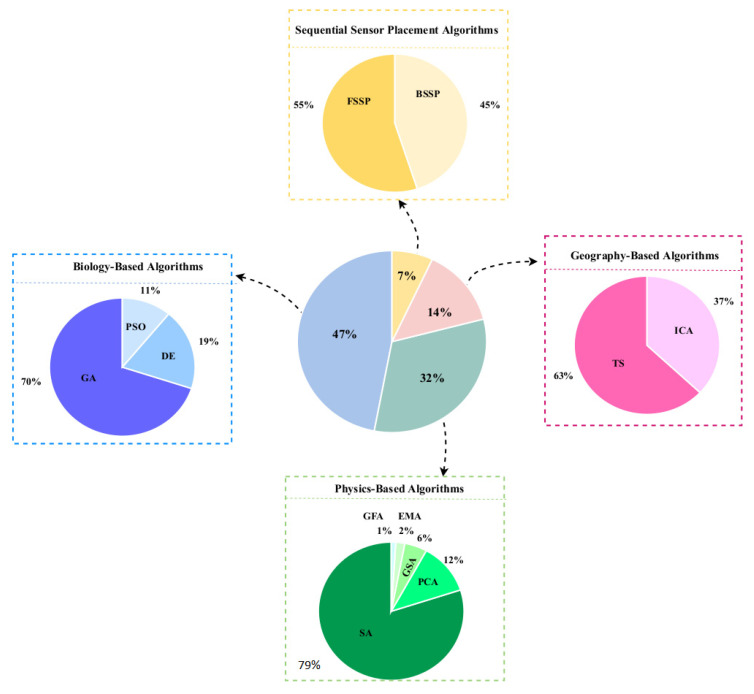
Pie charts of applications of optimization algorithms in SHM.

The optimization problem for OSP strategies is defined with the sensor locations being the discrete optimization variables (parameters), while the constraint is usually the number of sensors. The objective function is then minimized or maximized based on the dynamic characteristics of the structural system. Optimization methodologies used for OSP systems are reviewed in [35,36]. In the following sections, we present four popular optimization techniques used in OSP and SHM systems, including biology-based algorithms, geography-based algorithms, physics-based algorithms, and sequential placement algorithms. Figure 10 shows the percentage of use of each optimization algorithm in SHM (according to a keyword search on Google Scholar on 20 February 2023). As it turns out, biology-based algorithms are much more common.

### 4.1. Biology-Based Algorithms

Biology-based, or bioinspired, optimization algorithms are called memetic algorithms, due to their analogy with biological evolution and activity. The algorithms can be subdivided into two categories: trajectory-based algorithms and population-based algorithms. Population-based algorithms are also referred to as evolutionary algorithms (EAs). EAs are search methods that mimic biological evolution processes and species’ social behaviors. The subsequent generation surpasses the parents of algorithms through learning, evaluation, and adaptation. Among them, GA and its variations are the most common algorithms.

**Figure 11:**
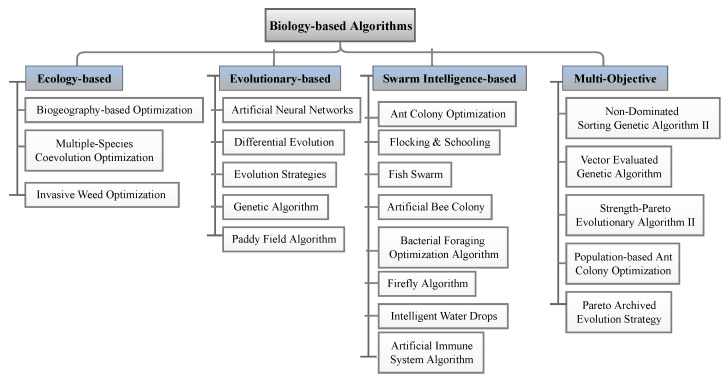
Classification of biology-based algorithms.

Various biological processes, in regard to the collective behavior of animals and natural evolution, have inspired the development of biology-based algorithms. These methods can be classified into two groups: swarm-based algorithms and evolution-based algorithms. Several evolutionary algorithms have been developed, including DE, GA, evolutionary strategies, cultural evolution, and genetic programming. This article divides these methods into four main groups: (I) ecology-based, (II) evolutionary-based, (III) swarm intelligence-based, and (IV) multi-objective algorithms. An overview of the classification of biology-based algorithms is presented in Figure 11.

The following provides details of the three most popular evolutionary-based algorithms used for SHM and OSP, i.e., Genetic Algorithm, Differential Evolution, and Particle Swarm Optimization. These algorithms are suitable for single-stage and two-stage methods with single-objective and multi-objectives.

#### 4.1.1. Genetic Algorithm (GA)

GA is based on Darwin’s theory of selection and evolution [213]. The features used in this optimization are genes, chromosomes, and population. A gene is an individual characterized by a set of parameters (variables). A chromosome is formed by joining genes into a string (solution). A population is composed of individuals who are the starting point of the process.

GA involves four main operations, e.g., encoding, selection, crossover, and mutation, as described below:

- Encoding: The problem’s input parameters, or decision variables, are encoded into a solution series of a finite length. Encoding methods include octal encoding, binary encoding, hexadecimal encoding, value encoding, permutation encoding, and tree encoding.
- Selection: In the selection stage, individuals are selected from a population for later breeding. Selection methods include rank selection, roulette wheel selection, Boltzmann selection, tournament selection, and stochastic universal sampling.
- Mutation: To introduce additional diversity, mutation randomly changes individuals. Mutations include displacement mutation, inversion mutation, scramble mutation, big flipping mutation, and reversing mutation.
- Crossover: During mating, a crossover point is randomly selected between each pair of parents. Crossover methods include K-point crossover, single-point crossover, partially mapped crossover, uniform crossover, order crossover, precedence preserving crossover, shuffle crossover, reduced surrogate crossover, and cycle crossover.

A step-by-step guide to implementing GA is provided below:

- Step 1: Set up GA parameters.
- Step 2: Create a random population of a specified size.
- Step 3: Calculate the objective function for all population members.
- Step 4: Select the best individuals from a population of candidates.
- Step 5: Perform crossover with two individuals, known as parents, randomly chosen from a mating pool to create two offspring.
- Step 6: Mutate the individuals of a population, based on mutation probabilities.
- Step 7: Perform elitism, where the best individuals in a generation are passed on to the next generation without undergoing any change.
- Step 8: Repeat steps 3 to 7 until the specified number of generations is reached, or the termination criterion is met.

**Figure 12:**
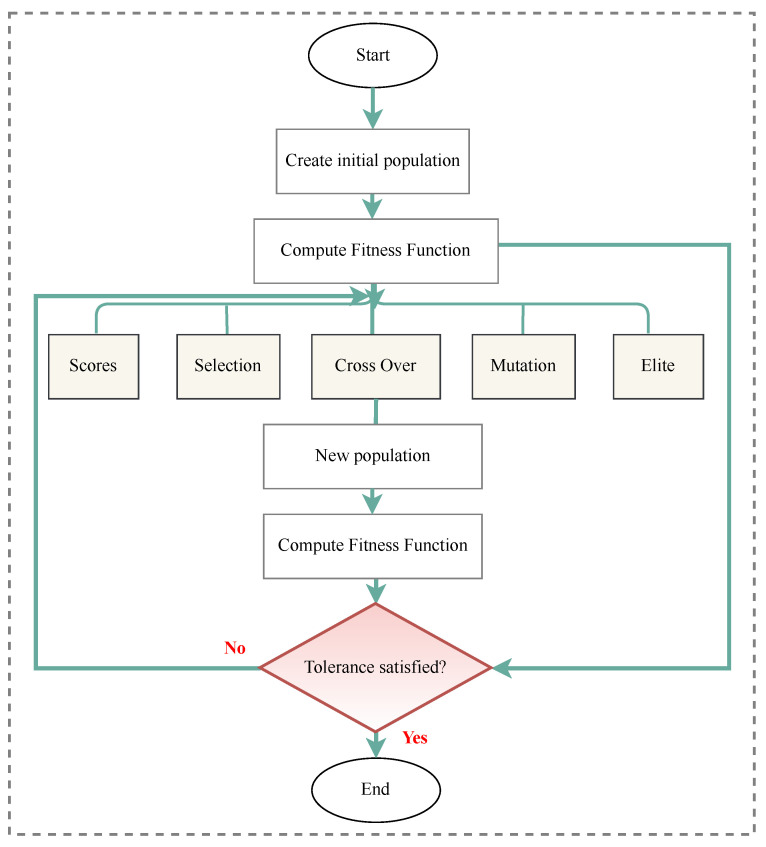
Flowchart of GA.

A flowchart for GA is presented in Figure 12, providing an overview of the algorithm. In addition, to help the reader understand the process, a pseudo-code for GA is presented using coding terminology for software such as MATLAB (see Algorithm 1). The advantages and disadvantages of GA are summarized in Table 13.
**Algorithm 1** Pseudo-code for GA.1:Determine Objective Function f(x).2:Assign the number of generations to 0 (t=0).3:Randomly create individuals in initial population P(t).4:Evaluate individuals in population P(t) using f(x).5:**while** Termination criterion is not satisfied **do**6:    t=t+17:    Select the individuals to population P(t) from P(t−1).8:    Change individuals of P(t) using crossover and mutation.9:    Evaluate individuals in population P(t) using f(x).10:**end while**11:Return the best individual found during the evolution.

GA is a heuristic method that solves many real-world and research problems, including engineering and classic optimization problems. Much recent work in the field of GA focuses on applying GA to multi-objective problems and using heuristic metamodels within GA to improve convergence time without sacrificing accuracy or usefulness. Four common types of GA are steady-state GA (SSGA), generational GA (GGA), (µ + µ)-GA, and steady-generational GA (SGGA). GAs, both original and improved, have been used to solve problems in OSP and SHM methods.

Liu et al. [214] proposed a novel approach to detect damage in a five-girder supported bridge using a support vector machine (SVM) optimized by a genetic algorithm. Using GA, the best kernel parameters were obtained from selection, mutation, and crossover and used as model parameters in SVM. A vector containing the frequency rate and mode shape ratio was used as the input variable. A maximum relative error of 1.84% was obtained by applying the GA–SVM algorithm for damage identification of a single damage case and multiple damage cases. In addition, GA–SVM was compared with the radial basis functions (RBFs) and backpropagation networks optimized by GAs (GA–BPs), achieving maximal relative error values of 6.91% and 5.52% for RBFs and GA–BPs, respectively.

Combining extended finite element modeling (XFEM) with GA is a highly effective method for detecting structural flaws. In this algorithm, XFEM models the forward problem, and GA is used for optimization. Convergence is achieved by minimizing the error between measurements obtained from sensors and data obtained from solving the forward problem. Chatzi et al. [215] proposed an improved XFEM–GA algorithm by accelerating the GA convergence, preventing entrapment in local optima, and formulating a generic XFEM to detect damage of any shape. The researchers verified their method experimentally on a 2D plate with arbitrary damage.

To solve the inverse problem of SHM, Yu et al. [216] proposed an FGAPSO algorithm, which is a fusion of GA and PSO algorithms. In order to improve the simple GA, which has the limitations of easy precociousness and low computational efficiency, a real-coded GA was developed. Chaotic logistic mapping was used to initialize the population, and crossover-mutation operators and elitist strategies were applied. Compared to conventional GAs and PSOs, the proposed algorithm proved more effective in detecting damage on a 13-bar truss structure.

The coverage and connectivity of target-based wireless sensor networks (WSNs) are the two most important factors for data forwarding from a target to a remote base station (BS). Finding a minimum number of possible positions to place sensor nodes that satisfy coverage and connectivity is a nondeterministic polynomial (NP). and a complete problem is given a set of target points. Gupta et al. [217] proposed an improved GA-based solution to this OSP problem. A fitness function was derived along with the usual GA operators and an efficient chromosome representation. The efficacy of the proposed scheme was demonstrated by comparing it with some related existing algorithms.

An improved GA method was developed by Beygzadeh et al. [44] for OSP in space structure damage detection. The researchers proposed a numerical forward algorithm and Geometrical Viewpoint and GA (GVGA) to minimize the impact of noise on the optimization algorithm. The error function was considered as the standard deviation of elliptical noise diameters in the space of response changes. This study applied GVGA and GA algorithms to the structures and compared the OSP results. The results showed that GVGA improved the algorithm’s convergence, leading to a better sensor pattern.

Ganesan et al. [218] proposed a novel GA employing a second-generation wavelet transform (SGWT) to identify optimal node placement locations. A bi-orthogonal Cohen–Daubechies–Feauveau wavelet (CDF5/3) was used to enhance the quality of the population matrix, and CDF5/3 filter-based lifting schemes were investigated to adjust the sensor positions. The algorithm was implemented to determine sensor positions with various populations. The novel method was compared to GA, random deployment, and GA with CDF5/3 wavelets and found to be superior.

#### 4.1.2. Differential Evolution (DE) Algorithm

DE is a population-based metaheuristic search algorithm that iteratively improves a candidate solution based on an evolutionary process. This algorithm was first presented by Storn and Price [219] in 1997. There are three real control parameters in this optimization algorithm: (1) crossover constant $C_{r}$, (2) differentiation constant *F*, and (3) population size. Other parameters include:

- stopping criteria that determine the maximum number of generations *G* (or iterations),
- the dimensions of problem *S* that scales the difficulty of optimization,
- the boundaries that limit the feasible area $X_{m}in$ and $X_{m}ax$.

In DE, the initial solution to the problem is generated as a set of random populations. A mutant vector $v_{i,m}$ is derived from three randomly selected target vectors. The mathematical representation of this process is:

(11) $$\nu_{i,m}=x_{i,3}+F\left(x_{i,1}-x_{i,2}\right)$$

The step-by-step procedure of DE is presented below:

- Step 1: Set up the DE parameters that are required for the algorithm.
- Step 2: Create a random population of the specified size.
- Step 3: Calculate the objective function for all solutions.
- Step 4: Select three different target vectors.
- Step 5: Determine the trial vector using the crossover constant.
- Step 6: Choose a vector between the trial and target vectors.
- Step 7: Repeat procedures 3 to 6 until the specified number of generations is reached.

**Figure 13:**
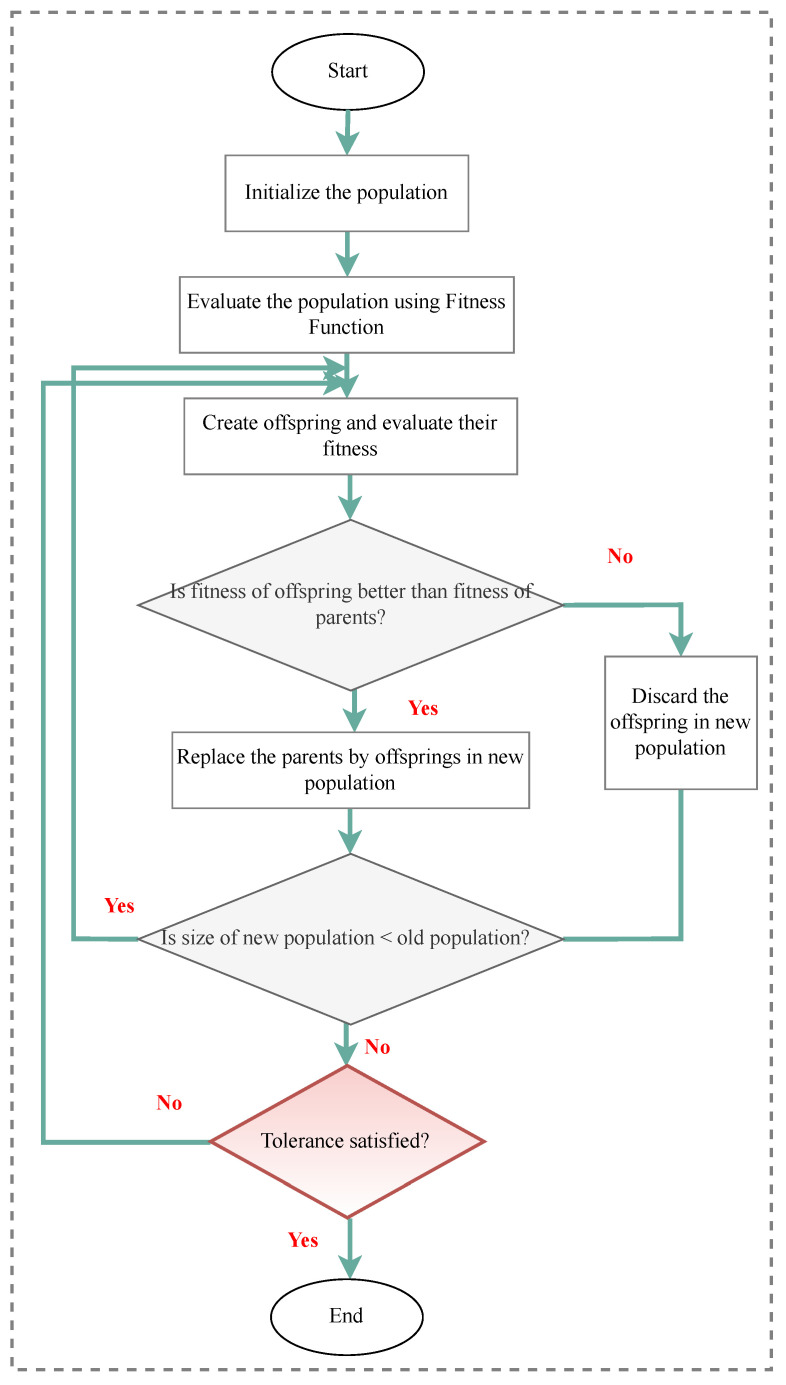
Flowchart of DE algorithm.

Figure 13 presents the flowchart of the DE algorithm, and Algorithm 2 provides the pseudo-code using coding terminology to assist the reader in understanding the process. The advantages and disadvantages of the DE algorithm are also summarized in Table 13.
**Algorithm 2** Pseudo-code for DE Algorithm.1:Initialize the population *x* with randomly generated solutions2:Set the weight $F\in \left[0,2\right]$ and crossover probability $C_{r}\in \left[0,1\right]$3:**while** stopping criterion **do**4:    **for** i=1:n **do**5:        For each $x_{i}$, randomly choose 3 distinct vectors $x_{p}$, $x_{r}$ and $x_{t}$6:        Generate a new vector ν by DE scheme7:        Generate a random index $J_{r}\in \left\{1,2,...,d\right\}$ by permutation8:        Generate a randomly distributed number $r_{i}\in \left[0,1\right]$9:      **end for**10:    **for** j=1tod **do**11:        For each parameter $\nu_{i,j}$ (*j*th component of $\nu_{i}$), update12:        $u_{i,j}^{t+1}=\left\{\begin{array}{c} \nu_{i,j}^{t+1}\mathrm{if}\;r_{i}⩽C_{r}\;\mathrm{or}\;j=J_{r}\\ x_{i,j}^{t^{\prime }}\mathrm{if}\;r_{i}>C_{r}\;\mathrm{and}\;j\neq J_{r}, \end{array}\right.$13:    **end for**14:    select and update the solution15:**end while**

DE algorithms have been studied to solve SHM and OSP problems. As such, Kim et al. [220] proposed an efficient technique for determining multiple damage locations and severity in truss structures based on the DE algorithm and vibration data. Natural frequencies and mode shapes were used to formulate the objective function. Three numerical examples of planar and space truss structures were examined to verify the effectiveness and practicality of this application. The DE-based method proved reliable for determining multiple damage conditions by comparing the proposed method with a GA approach.

Recently, a sine–cosine algorithm was investigated as a potential meta-heuristic method for structural damage detection. Bureerat and Pholdee [221] proposed integrating the algorithm’s leading reproduction operators with the mutation operator of DE to design a self-adaptive algorithm. Combined with the differential evolution (ASCA–DE) algorithm, this adaptive sine–cosine algorithm performed better than some established meta-heuristics.

A nonlinear optimization problem was proposed by Guedria et al. [222] based on an accelerated DE (ADE) algorithm for detecting and quantifying damage in plate-like structures. An objective function was established by altering the flexibility matrix of the structure with a penalty function that prevents false alarm detection. In the new ADE algorithm, three modifications were introduced to the standard DE algorithm, as follows:

(i) The initial population of a structure was generated using its damage scenario.
(ii) During the mutation process, a new difference vector was generated, based on the dispersion of individuals through the search space, to automatically balance local and global searching.
(iii) A new exchange operator was designed to avoid the untimely convergence of local optima.

The ADE method was validated in terms of solution accuracy and computational cost, as well as its ability to locate and assess damage even when the data was contaminated with noise.

Using an improved DE algorithm, Seok et al. [223] proposed a sensor deployment method for radio frequency identification (RFID) sensor networks for mobile robot localization. For surveillance and security, this study suggested two optimization strategies: direct optimization, which optimizes initial information intuitively, and full coverage optimization, which optimizes dense coverage. Experimental results showed that guided parameter settings resulted in better sensor deployment. In addition, the complete coverage optimization strategy also yielded excellent results based on guidelines from the standard DE algorithm.

Localization of sensor networks based on connectivity can be modeled as a nonconvex optimization problem. In current models, only convex constraints are considered, i.e., connections between the nodes. An algorithm based on heuristics and a modified DE algorithm was proposed by Qiao et al. [224] in the context of unknown communication ranges. The algorithm included a new crossover procedure to create a new generation of individuals/candidates. A “single node treatment” procedure was developed as part of the search procedure to construct a new set of location coordinates to jump out from the local minimum. Compared to other convex–constraint methods, the results indicated that better solutions could be achieved with the new method.

Wireless sensor networks (WSNs) with 3D directional sensor nodes are becoming increasingly popular in real-life applications, motivating research that optimizes sensor deployment. In [225], Cao et al. devised an optimization strategy for 3D directional WSNs by considering coverage, sensor node connectivity, lifetime, cluster header connectivity, and reliability. Based on a cooperative coevolutionary framework, a modified DE algorithm was proposed, based on crossover rate sort and polynomial-based mutation. Experimental results revealed the improved performance of the modified algorithm regarding optimization results and operation time.

#### 4.1.3. Particle Swarm Optimization (PSO) Algorithm

In 1995, Kennedy and Eberhart proposed an evolutionary computation method called PSO [189]. In this algorithm, every solution is considered a bird flock particle. Accordingly, in addition to individual intelligence, the birds show extended social behaviors and coordinate their movement to a specific goal. Initially, random positions and velocities are assumed for each particle in the search space. To find the optimal solution, the position and velocity of each particle are updated, based on three main factors:

1. $P_{B}est$ (best position): the best position of an individual particle.
2. $G_{B}est$ (global best position [Global-PSO]): the position of each particle is influenced by the best-fit particle in the entire swarm.
3. $L_{B}est$ (local best position [Local-PSO]): the position of each particle is influenced by the best-fit particle chosen from its immediate neighbors.

To find the best solution through iterations, the process begins by randomly generating a swarm of particles. The positions of the $i^{\mathrm{th}}$ particle in an *S*-dimensional search space provide a candidate solution for the optimization, where *S* is defined as the number of variables involved in the optimization problem. The $i^{\mathrm{th}}$ particle is calculated numerically using the following three vectors:

(i) Current position $X_{i}\left(t\right)=\left(x_{i1},x_{i2},\ldots ,x_{is}\right)$
(ii) Best previous position $Y_{i}\left(t\right)=\left(y_{i1},y_{i2},\ldots ,y_{is}\right)$
(iii) Flight velocity $V_{i}\left(t\right)=\left(v_{i1},v_{i2},\ldots ,v_{is}\right)$.

A particle moves to a new position during the search process finding $P_{\mathrm{best}}$ in each iteration. The neighbors of (($L_{\mathrm{best}}$)) are considered as best solutions, where

$$\begin{array}{c} P_{\mathrm{best}}=\left(p_{\mathrm{best},i1}p_{\mathrm{best},i1}\ldots p_{\mathrm{best},is}\right),\\ L_{\mathrm{best}}=\left(l_{\mathrm{best},i1}l_{\mathrm{best},i1}\ldots l_{\mathrm{best},is}\right) \end{array}$$

The velocity and position of each particle are updated using the following equations:

(12) $$\begin{array}{c} v_{ij}^{t+1}=w^{t}v_{ij}^{t}+c_{1}r_{1}\left(p_{\mathrm{best},ij}-x_{ij}^{t}\right)+c_{2}r_{2}\left(l_{\mathrm{best},ij}-x_{ij}^{t}\right) \end{array}$$

(13) $$\begin{array}{c} x_{ij}^{t+1}=x_{ij}^{t}+v_{ij}^{t}\;,\;\forall i\in P\;,\mathrm{and}\;\forall j\in S \end{array}$$

The parameters $r_{1}$ and $r_{2}$, in Equation (12), are two independent random numbers between 0 and 1. $c_{1}$ and $c_{2}$ are two positive constants named learning rates or factors. *w* is a factor of inertial weight at *t*th iteration to control the impact and calculated as

(14) $$w^{t}=w_{\max}-\frac{(w_{\max}-w_{\min})\times t}{t_{\max}}$$

where $w_{\min}$ and $w_{\max}$ are defined, respectively, as the initial inertial and final weight. *t* and $t_{\max}$ are the current iteration number and the maximum number of iterations. The velocity of the particles is an important factor that defines the resolution. Therefore, the velocity of each particle is usually limited between the range $\left[-v_{\max},v_{\max}\right]$. $v_{\max}$ is calculated as

(15) $$v_{\max}=\gamma \left(x_{\max}-x_{\min}\right)$$

where $x_{\max}$ and $x_{\min}$ denote the dynamic range of the variable in each dimension. The algorithm terminates after satisfying the convergence criteria or reaching the maximum number of iterations.

A step-by-step explanation of PSO is provided below:

- Step 1: Set up the PSO parameters needed for the algorithm.
- Step 2: Generate a random population of the specified size.
- Step 3: Calculate the objective function for each member of the population.
- Step 4: Update each particle’s velocity.
- Step 5: Update the particle positions.
- Step 6: Calculate the objective function for all particles.
- Step 7: Using elitism, the best-obtained results are saved.
- Step 8: Repeat steps 4 to 7 until the specified number of generations is met or a termination criterion is reached.

**Figure 14:**
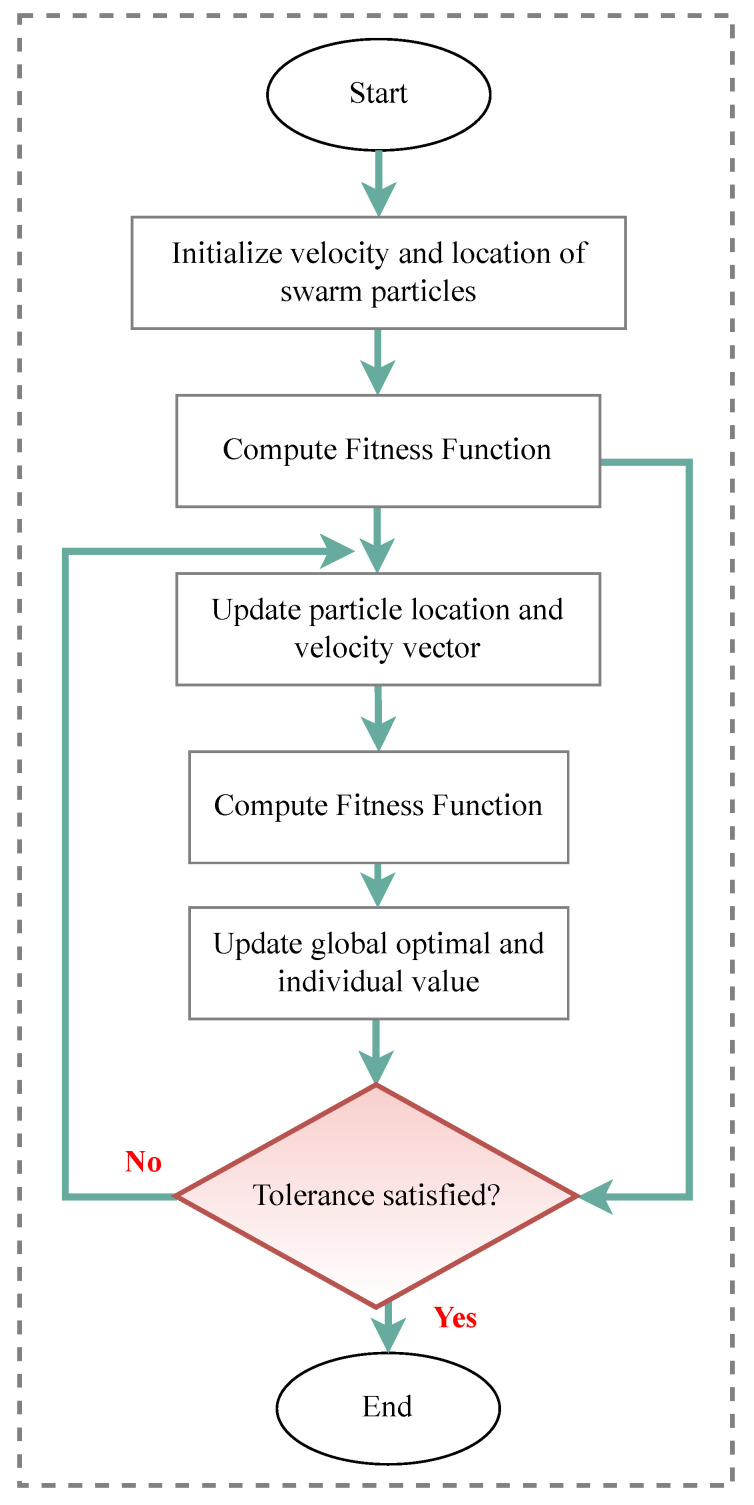
Flowchart of PSO algorithm.

The flowchart of PSO is shown in Figure 14, and Algorithm 3 presents the corresponding pseudo-code. The advantages and disadvantages of the PSO algorithm are summarized in Table 13.

Many advances in the PSO algorithm have been developed over the years that can be divided into six categories:

- Modifications of PSO, such as chaotic PSO, quantum-behaved PSO, and fuzzy PSO.
- Extensions of PSO to other optimization fields, such as multi-objective, discrete, constrained, and binary optimization.
- Hybridization of PSO with other metaheuristic methods, such as artificial immune system (AIS), GA, TS, and ACO.
- Parallel implementation of PSO, such as GPU computing, multicore, and cloud computing.
- Theoretical analysis of PSO, such as convergence analysis, and parameter selection.

**Algorithm 3** Pseudo-code for PSO Algorithm.1:Determine objective function f(x).2:Initialize parameters c1, c2, $w_{\max}$, $w_{\min}$, and population size nPop.3:Evaluate the fitness of each particle and set all initial positions as $P_{\mathrm{best},x}$.4:Generate an initial population of particles.5:**while**t<MaxGeneration**do**6:    Select the $G_{\mathrm{best}}$ particle in the swarm, which has the minimum fitness value7:    **for** i=1:nPop **do**8:        Calculate the velocity of particle $x_{i}$.9:        Update the position of particle $x_{i}$.10:    **end for**11:    **for** i=1:nPop **do**12:        Evaluate the fitness of updated particle $x_{i}$.13:        **if** $f\left(x_{i}\right)<f\left(P_{\mathrm{best},x}\right)$ **then**14:           Set current position as $P_{\mathrm{best},x}$.15:        **end if**16:    **end for**17:    Find the best particle18:**end while**

Both original and advanced PSO algorithms have been used for OSP and SHM problems. Chen et al. [226] proposed a new algorithm combining the PSO algorithm with an improved Nelder–Mead method, called the PSO–INM algorithm, to solve multi-sample objective functions based on Bayesian inference. Multi-sample objective functions provided stable patterns under various noise levels. An evaluation of the proposed method on a two-story frame structure demonstrated that it is sensitive to multi-damage cases.

In [227], a new hybrid PSO (HPSO) damage detection strategy was proposed, and its solution was studied using Monte Carlo simulations. First, Monte Carlo simulations tested the PSO algorithms with various parameters to determine which combination of parameters was most effective for damage identification. Following this, a robust local search Nelder–Mead algorithm was incorporated into the PSO. This strategy significantly improved the global search capability of the PSO, as verified by numerical and experimental tests.

Kaveh and Maniat [228] developed an innovative method using Magnetic Charged System Search (MCSS) and PSO to identify the location and extent of multi-damage in structures. A penalty approach was applied to moderate the effect of noise on the measured data. The proposed strategy was able to identify damage scenarios reliably and accurately, despite incomplete data and noisy measurement conditions.

A novel improved PSO (IPSO) algorithm was proposed by Zhang et al. [229] to address OSP problems. The modal number was initially selected using the cumulative effective modal mass participation ratio. In order to improve the PSO algorithm, three strategies were adopted, and the IPSO algorithm was applied to determine the most suitable sensor number and configuration. The proposed algorithm and four different PSO algorithms were evaluated in a latticed shell model case study. The PSO algorithms achieved satisfying OSP schemes, while the IPSO algorithm improved convergence speed and precision.

With the rapid development of the Internet of Things (IoT), intelligent homes and environments are becoming mainstream assets. Motion sensors are common features in automated environments, and their optimal placement ensures optimized coverage of an area with the fewest sensors. Here, two main challenges are finding the correct number of motion sensors and their locations. An algorithm combining whale optimization algorithm (WOA) and PSO was studied by Nasrollahzadeh et al. [230] to determine the optimal placement of motion sensors in smart homes. This hybrid algorithm was compared to previous methods and resulted in improved detection accuracy, coverage percentage, and operating costs.

A modified version of PSO for high-dimensional optimization problems is sequential PSO (S-PSO), as proposed by Ngatchou et al. [231]. Instead of optimizing the entire parameter space in a single step, S-PSO iteratively optimizes a subspace of the parameter search space each time. An S-PSO method was applied to identify distributed sonar sensor placement, where dimensionality and computational complexity were issues. The simulations showed that S-PSO was more efficient and converged faster than standard PSO.

A comparison of three well-known optimization algorithms (GA, PSO, and DE) is presented in Table 16. A significant amount of research has been published comparing the performance of these three evolutionary algorithms in solving some challenging optimization problems. Interested readers are referred to [232,233] for more information.

#### 4.1.4. Other Evolutionary Algorithms

In addition to the above-mentioned algorithms, various evolutionary algorithms have been studied for OSP and SHM problems. For instance, Sun et al. [147] solved the OSP problem using the ABC algorithm, miming honeybee foraging behavior. Hashim et al. [234] proposed an enhanced deployment algorithm based on ABC for optimal node placement in a wireless sensor network. Yi et al., in [235], developed a distributed wolf algorithm (DWA) for OSP problems with faster convergence and higher search capability, inspired by wolf behavior. Li et al. [236] proposed a multi-swarm fruit fly optimization algorithm (MFOA) to identify damage using modal data, such as the first few natural frequencies and mode shapes. In [200], Yi et al. developed a new optimum sensor array design method for SHM systems using a modified MA. Pan et al. [237] proposed a hybrid self-adaptive Firefly–Nelder–Mead (SA–FNM) algorithm to explore the SHM problem. In [238], a new evolutionary algorithm, the K-means Jaya, was used to train an ANN model to obtain optimal weights and biases by minimizing the discrepancy between actual and desired outputs.

### 4.2. Physics-Based Algorithms

Physics-based algorithms are heuristic algorithms that mimic matter’s physical properties or physical behavior. These algorithms include Simulated Annealing (SA), Gravitational Search Algorithm (GSA), Electromagnetism-Like Algorithm (EMA), Particle Collision Algorithm (PCA), and Gravitation Field Algorithm (GFA). Below, SA is discussed in more detail. The reader is referred to [239,240,241] for a comprehensive overview of other physics-inspired optimization techniques.

**Figure 15:**
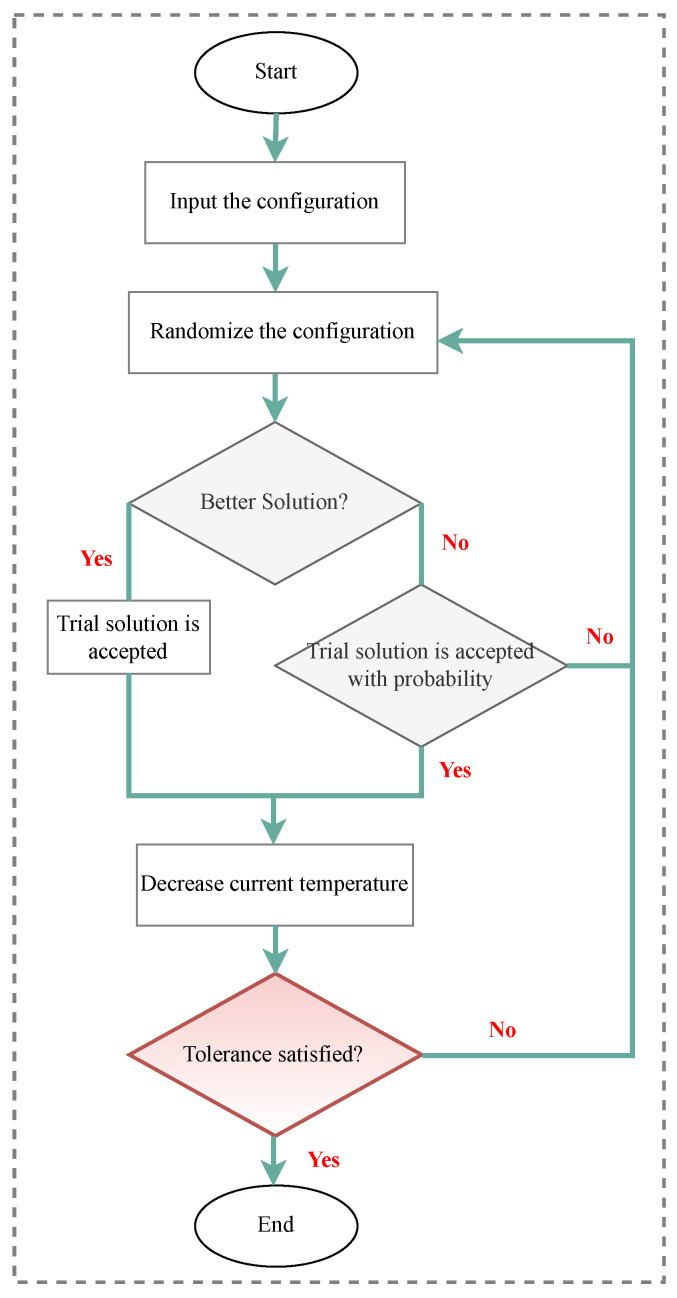
Flowchart of SA.

SA is a stochastic global search optimization algorithm inspired by annealing mechanisms in metallurgy. In the 1980s, SA significantly impacted the field of heuristic search for its simplicity and efficiency in solving combinatorial optimization problems. SA algorithms are based on the studies of Metropolis et al. [242] and Kirkpatrick et al. [243], who proposed an optimization process that simulates the thermodynamic physics of metal cooling and annealing [244]. Perturbation operators drive annealing in the iteration process. Every variable is perturbed randomly during the SA iteration. The operator uses direction cosines for each variable to generate a random direction. By using constraints, the search domain is kept within iterations until the best solution is found. A flowchart of SA is presented in Figure 15. The advantages and disadvantages of the algorithm are summarized in Table 13.

Applications of SA are numerous, and include SHM and OSP applications. As such, He et al. [245] developed an algorithm combining an adaptive real-parameter GA with SA to detect damage in beam-type structures. The finite element software ANSYS was used to obtain natural frequencies and static displacements. The proposed algorithm effectively identified flexural stiffness damage in beam-type systems in noise-free and noisy conditions.

An innovative method to detect damage in a self-anchored suspension bridge was introduced in [246]. Here, a BP neural network was first constructed to estimate the damage locations. Then, a genetic-SA algorithm was proposed using the characteristics of GA and SA algorithms to identify the locations and extent of the damage. The new algorithm achieved an improved global convergence effect over traditional GA by incorporating the Metropolis acceptance rule of the SA algorithm.

In [247], Zimmerman et al. proposed an innovative parallelization of the SA stochastic search algorithm to compare model predictions with experimental results and used this algorithm to update structural models. A three-story steel structure, subjected to seismic base motion, was tested using the resulting distributed model updating algorithm within a network of wireless sensors.

An improved SA algorithm was presented by Tong et al. [248] to solve a sensor placement problem. The algorithm was developed using the coordinate system of the sensor location to enable further dimension searching while minimizing computational efforts in SA’s random search. According to the results, the proposed method outperformed conventional SA and GA in the search for the optimal placement of sensors.

As reported in [249], a robust OSP framework was proposed by Nasr et al. by combining an optimization-based algorithm, SA, and ensemble Kalman filter (EnKF). The difference between the actual measured data and its corresponding EnKF predictions was used as an objective function. A comparison was conducted between the results and the optimal sensor locations determined by a brute-force search method.

The SA method was further used in conjunction with artificial neural networks (ANNs) to predict the response of reinforced concrete (RC) shear walls [250]. For system identification, Jeong et al. [251] proposed an efficient hybrid algorithm, named ASAGA (Adaptive Simulated Annealing Genetic Algorithm).

### 4.3. Geography-Based Algorithms

**Figure 16:**
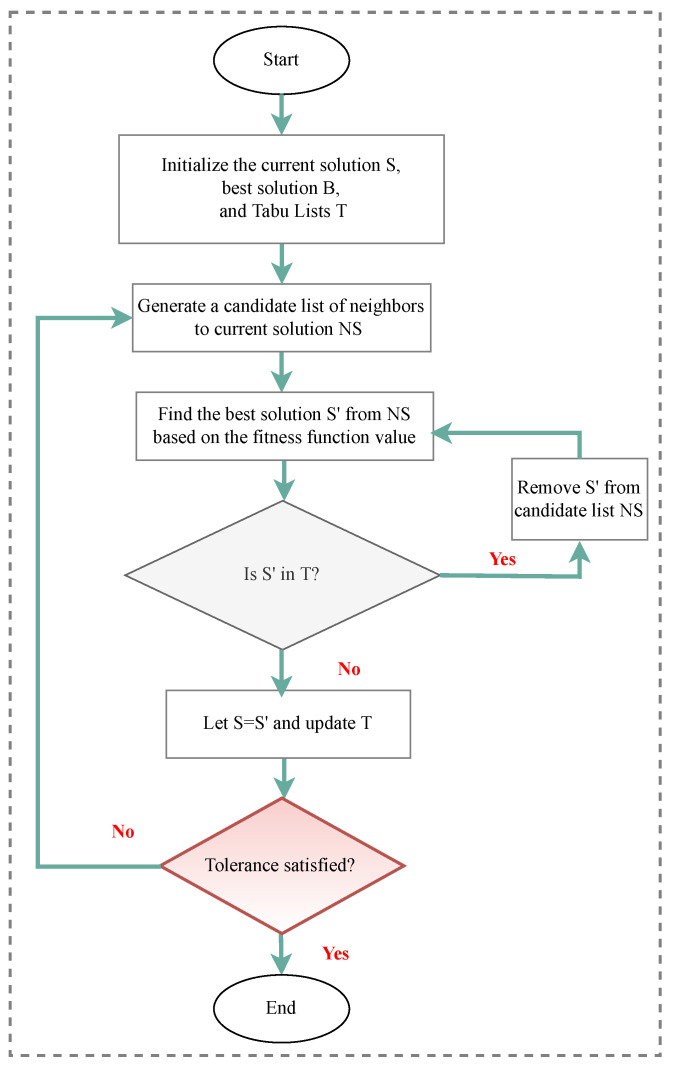
Flowchart of TS.

Geography-based optimization is a metaheuristic algorithm that generates random solutions in the geographical search space. The imperialistic competition algorithm (ICA) and Tabu Search (TS) are examples of these algorithms. A defining characteristic of TS is that it generates an updated list of solutions that are not allowed. Further, a critical factor is considered that relaxes the optimization criteria if no better solutions exist. In this way, local minima can be overcome. The TS flowchart is shown in Figure 16, and Table 13 summarizes the advantages and disadvantages of TS.

Arafa et al. [252] developed a hybrid optimization approach that included two components: modified continuous reactive TS (MCRTS) and real-coded GAs. The algorithm was tested on several beam structures with crack damage, and the natural frequencies were used as features. After several runs, the developed algorithm consistently found the two sought-for optima. In [253], a TS heuristic was proposed to solve both economic criteria and critical variables for the optimal design and upgrading of sensors. Two industrial process networks were compared for their performance using stochastic solution strategies. A TS-based routing algorithm (TSRA) was proposed by Orojloo et al. [254] for determining the optimal route from a source to a destination in WSNs. This algorithm integrated energy consumption and hopped counts into routing decisions using a new move and neighborhood search method. The TSRA obtained more balanced transmission among nodes, reduced energy consumption and routing costs, and extended the network’s lifetime for different randomly generated networks. It was shown in [255] that the TS algorithm could be used for the OSP problem in moving force identification. Some numerical simulations were conducted on a 2D planar truss model to evaluate a proposed TS-based OSP procedure. An optimization objective function was defined as the MAC matrix’s mean values of off-diagonal elements. The TS-based OSP approach was found to be feasible and more accurate compared to previous methods.

### 4.4. Sequential Sensor Placement Algorithms

In general, meta-heuristic algorithms use low computational power but require a long time to reach an optimal solution. Hence, they are designed to find a solution that is “good enough” in a time that is “small enough”. Likewise, sequential sensor placement (SSP) algorithms are computationally efficient but may require a long time to determine the true global optimum from the obtained solution. Iterative algorithms, such as SSP, optimize error functions by adding or removing a subset of candidates in each iteration. In cases where the candidates are sequentially removed, the algorithms are termed backward sequential sensor placement (BSSP), while in cases where they are added, they are termed forward sequential sensor placement (FSSP) [256].

Several works in the area of OSP in SHM are based on SSP algorithms. For instance, a novel relaxation sequential (RS) algorithm was proposed by Yin et al. [257] to address the considerable tension in the optimal sensor placement solution obtained by the sequential algorithm. Here, the sequential algorithm was modified to incorporate Dijkstra’s edge relaxation algorithm. Using the sequential algorithm, an initial solution set was generated, which relaxation improved until the relaxation operation terminated. As an error function, the MAC was used in this algorithm. According to the results, the RS algorithm derived a solution with fewer sensors and could reach higher maximum off-diagonal elements in the MAC matrix.

In [258], Lam et al. developed an enhanced sequential sensor placement (ESSP) algorithm to address computational bottlenecks in candidate configurations with many DOFs. A minimum sensor interval and spatially corrected prediction errors were considered to address sensor redundancy in finely meshing models. Compared with conventional methods, the proposed method achieved superior sensor configurations.

While SSP methodologies are computationally efficient, since they have a deterministic number of computations, multi-objective optimization cannot easily be implemented. Due to this limitation, as well as the increase in available computing power, SSP algorithms have become obsolete. For real-world applications, all subsequent meta-heuristic algorithms outperformed SSP algorithms. The purpose of optimization algorithms is to achieve the most optimal results. According to the “no free lunch” theorem [259], the results of all optimization technique overall class problems are similar. Performance improvements for one class of problems results in performance drops for other types.

### 4.5. Other Optimization Methodologies

In addition to the optimization methodologies presented above, researchers developed and employed various other techniques to detect damage and find optimized sensor configurations. For instance, an optimization strategy called Snobfit [260] was designed by Guratzsch [19] to optimize noisy objective functions under bound constraints. In [261], a novel approach to detecting damage to trusses, space frames, and plate structures was presented by combining Bayesian data fusion with teaching–learning-based optimization (TLBO) algorithms. The Kalman filter algorithm [262], which estimates state vectors and the variances of errors, was utilized in combination with sequential placement strategies for addressing OSP problems in [263]. In [264], a forward sub-structuring approach, modal strain energy, and the enhanced bat algorithm (EBA) were utilized in an effective three-stage method for detecting damage in large-scale space structures. Additionally, a variety of other methodologies are also available for OSP, such as the following: energy-efficient sensor deployment [265], backup sensor-based fault-tolerance SHM method [266], mixed variable pattern search algorithm [267], frequency domain-based OSP technique [268], three-phase sensor placement approach [269], Gram–Schmidt orthogonalization procedure [270], e-Estimator algorithms [271], and wave propagation-based local interaction simulation approach [272]. SHM problems can also be analyzed using other computational methodologies, including the guided water strider algorithm [273], grasshopper optimization algorithm (GOA) [274], improved imperialist competitive algorithm [275], atom search algorithm (ASO) [276], equilibrium optimizer algorithm [277], grey wolf optimizer algorithm [278], balancing composite motion optimization [279], Q-learning evolutionary algorithm [280], Kriging-particle swarm optimization algorithm [281], and topology optimization [282]. More details of these methodologies can be found in the associated studies.

As mentioned earlier, optimization algorithms are widely used in OSP and at all levels of SHM. To provide an overview of optimization algorithm applications in these fields, the authors extensively reviewed related articles in Table 17. Table 18 and Table 19 provide an overview of the application of optimization algorithms in OSP and SHM methods, respectively. In these two tables, optimization algorithms and corresponding objective functions are listed.

### 4.6. Using Optimization Algorithms in Artificial Intelligence

Optimization algorithms are further used in AI technologies, such as ANN [337], ML [338], and DL [339], to optimize and automatically improve the learning. As such, a neural network optimizer changes attributes of the network, such as weights and learning rate, to reduce losses. The first step in solving a neural network problem is to define a loss function. Optimization algorithms and strategies are used to minimize losses to provide the most accurate results. Different types of optimizers to reduce the loss function include the following: gradient descent, stochastic gradient descent (SGD), mini-batch stochastic gradient descent (MB-SGD), SGD with momentum, Nesterov accelerated gradient (NAG), adaptive gradient (AdaGrad), AdaDelta, RMSprop, and Adam.

Much research has recently focused on combining AI and OA for structural damage detection and optimized sensor placement. A novel technique for training ANNs in detecting laminated composite structural damages was proposed by Tran-Ngoc et al. [64] using gradient descent (GD) techniques and a global search capacity of EAs. Further, the researchers proposed a hybrid metaheuristic optimization algorithm (HGACS) to enhance global search efficiency by combining the advantages of GA and Cuckoo Search. For SHM of laminated composite plates, Shirazi et al. [66] developed a new hybrid YUKI–ANN. A modified ANN was used to predict damage levels using four optimization algorithms: Balancing Composite Motion Optimization (BCMO), Arithmetic Optimizing Algorithm (AOA), PSO, and YUKI algorithm. The study revealed superior YUKI algorithm (YA) results compared to the AOA, PSO, and BCMO algorithms. In [340], a flexible combination of an ANN and a Cuckoo Search (CS) algorithm was used to detect structural damage. ANNs used CS to improve the training parameters (weight and bias) by minimizing the difference between the actual and desired outputs. The authors in [341] addressed the application of the ANN-enhanced Jaya algorithm (ANN–E JAYA) in predicting tensile load reduction as a function of crack lengths from an extended FEM (XFEM).

Recently, Lan et al. [342] developed an Optimized AdaBoost–Linear SVM approach to predict bridge damage based on raw vibrational signals from passing vehicles. In this paper, an optimizing algorithm was designed to modify its configuration to make Linear SVM an effective component learner in AdaBoost. The authors in Yu et al. [343] proposed a hybrid framework for structural damage diagnosis based on principal component analysis (PCA), deep stacked autoencoders (DSAE), and data fusion. To improve the diagnosis model, the authors optimized the meta-parameters of DSAE using the enhanced whale optimization algorithm (EWOA), which included the dropout parameter, the weight decay coefficient, the learning rate, and the hidden layer neuron numbers. An example of ANN techniques applying optimization algorithms to detect composite damage can be found in [344]. The results in this paper showed how recent optimization techniques have been used to calibrate the influential parameters during the training of ANN, including Jaya, Whale Optimization Algorithm (WOA), enhanced Jaya (E-Jaya), and Arithmetic Optimization Algorithm (AOA).

## 5. Conclusions

This study systematically reviewed the application of optimization algorithms (OAs) for structural health monitoring (SHM) and optimal sensor placement (OSP). The integration of optimization methodologies for monitoring strategies is essential for the design of cost-effective and reliable systems. The paper provided a comprehensive overview of current SHM and OSP techniques. It included problem definitions and critical discussions on the advantages and disadvantages of the principal methodologies and suitable applications. The state-of-the-art optimization algorithms were presented, including biology-based algorithms, physics-based algorithms, geography-based algorithms, and sequential placement algorithms. Since evolutionary algorithms outperform other methods when addressing combinational methodologies, such as OSP, the most widely used evolutionary algorithms, and their most recently improved variants were summarized. Using machine learning algorithms in SHM and OSP is an emerging technology with many advantages over traditional approaches. As a final note, the study discussed the suitability of computational methods for specific OSP and SHM applications, where evolutionary algorithms are used more commonly. Numerous summary tables were provided as a guide for the reader to solve SHM and OSP problems using the most suitable optimization methodology.

## Figures and Tables

**Table 1:** Damage identification levels.

Level	Definition
Level I	Detection: Qualitative indication of damage presence
Level II	Localization: Estimation of damage position
Level III	Classification: Determination of damage type
Level IV	Quantification: Assessment of damage extent
Level V	Prognosis: Estimation of the remaining useful life of the system

**Table 2:** Review papers on optimization algorithms for SHM systems (between 2010–2023).

Ref.	Year	Journal	Description
Hart and Murray [30]	2010	Journal of Water Resources Planning and Management	This paper reviewed recently proposed optimization-based sensor placement strategies in SHM systems for water distribution systems.
Gupta et al. [31]	2010	Journal of Intelligent Material Systems and Structures	This article presented various optimization criteria used by researchers for the optimal placement of piezoelectric sensors and actuators on an intelligent structure.
Yi and Li [32]	2012	International Journal of Distributed Sensor Networks	This paper reviewed current developments and research on OSP systems from the viewpoint of both engineers and researchers.
Noel et al. [33]	2017	IEEE Communications Surveys & Tutorials	This paper evaluated SHM strategies using wireless sensor networks (WSNs), providing an overview of current algorithms used for damage detection and localization, as well as outlining challenges associated with network design and future research directions.
Adedoja et al. [34]	2018	Urban Water Journal	An overview of the state-of-the-art of OSP in a water distribution network was presented in this paper, as well as possible solutions and future research directions.
Ostachowicz et al. [35]	2019	Structural Health Monitoring	This article presented a definition of the optimization problem for SHM systems and an overview of optimization strategies for sensor placement.
Sony et al. [12]	2019	Structural Control and Health Monitoring	In this work, next-generation smart sensing technologies, including smartphones, UAVs, cameras, and robotic sensors, were reviewed for their application in vibration-based SHM.
Tan and Zhang [36]	2020	Structural Health Monitoring	This study comprehensively reviewed computational methodologies, such as optimization techniques, to optimize the sensor placement in SHM systems.
Barthorpe and Worden [37]	2020	Journal of Sensor and Actuator Networks	This paper reviewed advancements in the design of SHM systems, from sensor placement optimization (SPO) strategies to system evaluation.
Hassani et al. [38]	2022	Sensors	This study presented an overview of current developments in sensing technologies, sensor placement, and damage detection for composite structures.
Ghannadi et al. [39]	2023	Frattura ed Integrità Strutturale	Simulation annealing algorithms were examined in this work for various SHM applications, including damage detection, optimal sensor placement, and updating of finite element models.

**Table 3:** Papers on optimization algorithms used for OSP (between 2010–2023).

Optimization Algorithm	OSP Method	Year	Ref.
GA	Bayesian statistics approach	2010	[40]
PSO algorithm	Fisher information matrix	2011	[41]
Single parenthood GA (SPGA)	Modal assurance criterion	2012	[42]
Improved discrete PSO (IDPSO)	Nearest neighbor index	2013	[43]
Geometrical viewpoint and GA (GVGA)	Modal assurance criterion	2014	[44]
GA-based evolutionary optimization	Modal assurance criterion	2015	[45]
Stochastic optimization	Bayesian experimental design approach	2016	[46]
GA	Effective independence method	2017	[47]
Jaya algorithm	Reduced order model	2018	[23]
Quantum-inspired evolutionary optimization algorithm (DQEA)	Triaxial modal assurance criterion	2019	[48]
PSO algorithm	Multi-objective decision-making strategy	2020	[49]
GA	Iterative updating process	2021	[50]
Multi-objective optimization algorithm	Effective independence method	2022	[51]
GA	Augmented Kalman Filter (AKF) technique	2023	[52]

**Table 4:** Papers on optimization algorithms used for SDD (between 2010–2023).

Optimization Algorithm	Analysis Type	Damage Type	Data Type	Monitored System	Year	Ref.
Real-coded parallel GA	Model-based method	Crack	Operational modal data	Reinforced concrete beam	2010	[53]
Modified GA (MGA)	Efficient correlation-based index	Stiffness reduction	Natural frequency	Cantilevered beam	2011	[54]
Immunity-enhanced PSO (IEPSO) algorithm	Inverse problem	Stiffness reduction	Natural frequency and mode shape	Beam and truss	2012	[55]
PSO algorithm	Model-based method	Stiffness reduction	Frequency response function (FRF)	Beam and plane frame	2013	[56]
Hybrid algorithm of GA and PSO	Sensitivity-based analysis	Reduction of modulus of elasticity	Natural frequency and mode shape	Laminated composite beam	2014	[57]
Hybrid multi-objective GAs (NS2-IRRGAs)	Inverse problem using modal strain energy (MSE)	Stiffness reduction	Mode shape and stiffness matrix	3D steel structure	2015	[58]
Democratic PSO (DPSO) algorithm	Modal assurance criterion (MAC) and flexibility matrix	Crack	Modal data	Five-story shear frame	2016	[59]
Modified DE (MDE) algorithm	Flexibility-inverse problem	Delamination	Modal data	Composite plate	2017	[60]
Heuristic optimization (GA)	Model updating problem	Circular hole and delamination	Natural frequency	CFRP plate	2018	[61]
Sunflower optimization (SFO) algorithm	Multi-modal-inverse problem	Crack	Modal data	Composite plate	2019	[62]
Grey wolf (GW) optimization and Harris hawks (HH) optimization	Residual force vector	Crack	Expanded mode shape	Cantilever beam and truss tower	2020	[63]
Hybrid metaheuristic optimization algorithm (HGACS)	ANN-based method	Delamination	Modal data	Laminated composite structure	2021	[64]
PSO algorithm	Sensitivity-based method	Stiffness reduction	Mode shape	3D truss	2022	[65]
YUKI-ANN algorithm	Modal strain energy	Stiffness reduction	Modal data	Laminated composite plates	2023	[66]

**Table 5:** Exclusion and inclusion criteria in selecting the reviewed articles.

Inclusion Criteria	Exclusion Criteria
Titles, abstracts, or keywords include the following search keywords: “Optimization Algorithm” “Structural Health Monitoring” “Optimal Sensor placement”	Studies published before 2000. Duplicated papers (only one paper included). Articles unrelated to optimization algorithms. Non-English papers. Papers not peer-reviewed. Poor quality papers.

**Table 6:** Overview of reviewed journals and the covered content, i.e., optimization algorithms (OA), SHM systems, and OSP methods.

Journal	Founding Year	Best Quartile	OA	SHM	OSP
Ultrasonics	1963	Q1	721	358	28
Journal of Sound and Vibration	1964	Q1	2368	2812	310
Meccanica	1966	Q2	424	75	22
Engineering Structures	1970	Q1	2059	2589	190
Optical Fiber Technology	1970	Q2	470	190	16
Computers and Structures	1971	Q1	2707	171	78
Networks	1971	Q1	1349	36	351
Mathematical Programming	1971	Q1	4043	2	11
Computers and Operations Research	1974	Q1	4861	11	23
Mathematics of Operations Research	1976	Q1	8928	434	6672
International Journal of Remote Sensing	1980	Q1	2424	211	2211
Composite structures	1983	Q1	2148	683	123
Algorithmica	1986	Q1	1363	4	21
Mechanical Systems and Signal Processing	1987	Q1	3365	1552	390
Neural Networks	1988	Q1	2688	19	28
Engineering Applications of Artificial Intelligence	1988	Q1	3607	161	85
Neurocomputing	1989	Q1	11,095	157	106
Expert Systems with Applications	1990	Q1	10,502	325	141
Computational Optimization and Applications	1992	Q1	1907	6	12
Optimization Methods and Software	1992	Q1	1648	35	220
Remote Sensing	1992	Q1	1197	400	123
Mathematical Problems in Engineering	1992	Q2	1584	372	758
Journal of Combinatorial Optimization	1997	Q2	1827	9	20
Advances in Structural Engineering	1999	Q2	343	302	234
Optimization and Engineering	2000	Q2	847	20	15
Structural and Multidisciplinary Optimization	2000	Q1	3889	69	92
Structural and Multidisciplinary Optimization	2000	Q1	3889	170	92
Sensors	2001	Q1	3178	1708	100
Applied Soft Computing	2001	Q1	7230	166	128
Structural Health Monitoring	2002	Q1	633	4370	550
Discrete Optimization	2004	Q2	605	2	4
Structural Control and Health Monitoring	2004	Q1	842	1385	649
Cluster Computing	2005	Q2	2022	62	102
Measurement	2010	Q1	4000	289	939

**Table 7:** Types of measurements and sensors.

Measurement Type	Sensor Type
Velocity	Magnetic induction, Piezoelectric, Optical
Displacement	Inductive, Capacitive, Gyroscope, Optical, Magnetic, Acoustic emission, Ultrasonic
Acceleration	Capacitive, Piezoelectric, MEMS, Piezoresistive
Force	Optical, Piezoresistive
Strain	Optical, Piezoresistive
Pressure	Piezoresistive
Temperature	Acoustic, Thermoresistive, Optical, Thermoelectric

**Table 8:** Recent review papers on sensor systems for SHM.

Ref.	Year	Journal	Description
Wu et al. [105]	2020	Sensors	A comprehensive summary was presented on FOSs used for SHM, including detailed working mechanisms, categories, and principles of FOSs.
Dutta et al. [15]	2021	IEEE Sensors Journal	This paper reviewed recent developments of sensors for high-temperature SHM and advanced fabrication methods, such as fiber Bragg grating (FBG) sensors, eddy current sensors, and low-temperature ceramic technology.
Rocha et al. [13]	2021	Engineering Structures	This work reviewed the most common types of sensors used for laboratory and commercial applications of SHM for aerospace composites.
Mustapha et al. [106]	2021	Vibration	Sensor networks were reviewed for monitoring systems addressing various topics, including optimized sensor networks, force sensors, data transmission, information communication, and data analysis.
Grabowski et al. [107]	2021	Measurement	This paper presented the sensing capabilities of MXene nanomaterials for SHM, including two-dimensional nanomaterials with carbide or nitride layers (X layer) sandwiched between transition metal layers (M-layer).
Li et al. [108]	2022	Construction and Building Materials	This work presented graphene-based nanomaterials (GBNs) used as additives to cementitious materials to form self-sensing composites for SHM systems.
Glisic [97]	2022	Sensors	This paper presented a historical overview of the first hundred years of strain-sensing technology used for civil structure monitoring, outlining transformative milestones and possible future research directions.
Gao et al. [109]	2022	Applied Sciences	This work comprehensively presented recent research advances, challenges, and achievements of flexible piezoresistive strain sensors (FPSs) used for civil SHM.
Jayawickrema et al. [110]	2022	Measurement	Recent publications were reviewed on SHM systems for pipelines, buildings, and bridges, focusing on emerging FOS technology and the application of deep learning (DL) for advanced data analysis.
Hassani and Dackermann [8]	2023	Sensors	A systematic review of conventional and advanced sensor technologies was conducted in this article to provide input parameters for NDT and SHM systems and to determine whether they are suitable for determining the health state of structures.

**Table 9:** Recent review papers on SHM systems.

Ref.	Year	Journal	Description
Toh and Park [130]	2020	Applied Sciences	This review paper summarized studies applying ML algorithms for fault monitoring.
Azimi et al. [131]	2020	Sensors	This work comprehensively reviewed research on SHM concerning emerging DL-based methods and presented several SHM applications.
Flah et al. [132]	2021	Archives of Computational Methods in Engineering	This review comprehensively reviewed applications of various ML algorithms in SHM systems, including image-based SHM and vibration-based SHM.
Avci et al. [1]	2021	Mechanical Systems and Signal Processing	This paper thoroughly outlined gaps in SHM concerning conventional methods, and presented the most recent applications of DL and ML algorithms in damage detection based on vibration data for civil structures.
Mishra et al. [133]	2022	Journal of Building Engineering	This paper presented a review on SHM of civil engineering infrastructure, focusing on applications of the wireless Internet of Things (IoT)-based real-time wireless sensors technology.
Gordan et al. [134]	2022	Measurement	This work presented functions, models, and categories of data mining (DM) strategies, including GA, fuzzy logic, ANN, and principal element analysis, used for SHM systems.
Ramalho et al. [135]	2022	Structural Control and Health Monitoring	This article comprehensively reviewed testing procedures, equipment, and techniques adopted in NDT and SHM systems. It also presented the basics of Lamb waves and their application to fault identification, ML, statistical analysis, simulation methods, and signal processing.
Civera and Surace [136]	2022	Sensors	This work reviewed recent developments in NDT systems, including acoustic emissions, visual inspection, ultrasonic testing, radiographic testing, infrared thermography, oil monitoring, and electromagnetic testing.
Hassani et al. [38]	2022	Sensors	In this work, the authors comprehensively reviewed the development history of, and research in, different damage detection strategies in composite laminated plates.
Payawal and Kim [137]	2023	Applied Sciences	A review of image-based SHM applications was conducted, which includes discovering and identifying, monitoring and measuring, automating and improving efficiency, and promoting 3D model development.

**Table 10:** Recent papers presenting new data analysis methods for SHM.

Ref.	Year	Analysis Method	Monitored System	Description
Azimi et al. [131]	2020	Unsupervised deep neural network	Bridge	This paper proposed a damage detection technique using an unsupervised deep neural network, defined as a deep convolutional denoising autoencoder. In this method, multi-dimensional cross-correlation functions were used as input.
Choe et al. [138]	2021	LSTM	Wind turbine blade	This paper presented a technique concerning sequence-based modeling of DL using gated recurrent unit (GRU) neural networks and an LSTM algorithm to detect structural damage in floating offshore wind turbine (FOWT) blades.
Movsessian et al. [139]	2021	ANN	Wind turbine blade	This study presented a new ANN method that could establish non-linear relationships between particular damage-sensitive features affected by EOCs and new indicators using the Mahalanobis distance (MD).
Hassani et al. [75]	2022	EMD algorithm	Composite plate and spatial truss	This work proposed a new sensitivity-based model concerning the EMD algorithm to detect damage to systems with closely-spaced eigenvalues.
Corbally and Malekjafarian [140]	2022	Data-driven approach	Bridge	This paper presented a new data-driven strategy using ANNs to analyze acceleration records from multiple passes of a traversing vehicle for drive-by monitoring of bridges.
Hajializadeh [141]	2022	DL	Bridge	This paper proposed a novel numerical data-driven damage detection system using a deep convolutional neural network on train-borne signals while moving over a bridge at traffic speed.
Xu et al. [142]	2022	Bayesian method	Wind turbine blade	This paper proposed a time series analysis method, based on Bayesian cointegration, to include more than two damage-sensitive features in the analysis simultaneously.
Hassani et al. [74]	2022	VMD algorithm	Composite plate	In this work, a novel strategy was proposed using VMD algorithm to assemble a new set of input responses captured from condensed frequency response function rows for use in a model updating problem, based on sensitivity.
Mousavi et al. [143]	2022	Signal processing	Steel truss bridge	This work proposed a method based on the complete ensemble EMD algorithm with adaptive noise for identifying damage presence, location, and severity in a steel truss model of a bridge.
Hassani et al. [65]	2022	Model updating method	3D truss and composite plate	This work proposed a new optimization problem using a modal data-based sensitivity method for reliable and fast damage detection of systems with closely-spaced eigenvalues, such as 3D truss and composite structures.

**Table 11:** Recent papers on OSP methods.

Ref.	Year	Method	Description
Song and Jin [149]	2008	Sensitivity-based methods, EI and MAC	This work presented an optimization approach for sensor placement using eigenvector sensitivity, EI, and MAC methods.
Dinh-Cong et al. [150]	2019	MKE	This paper proposed a new two-stage method for sensor optimization and damage detection using symbiotic organisms search algorithm and modal kinetic energy change ratio.
Blachowski [151]	2019	Sensitivity-based methods	This study proposed an approach using a non-negative least square (NNLS) solution and sensitivity and norm minimization for OSP and damage detection in 3D truss structures.
Yang et al. [152]	2019	Redundancy elimination model	This work presented a novel redundancy elimination model that distributed global and local sensors based on the minor enclosing circle method and a sub-clustering algorithm.
Ariga et al. [153]	2020	Mutual information	This paper presented an OSP method, based on mutual information, using a Gaussian process (GP) and the sound-field-interpolation kernel for covariance measurements in a GP model to suitably place sensors.
Bhattacharyya and Beck [154]	2020	Mutual information	This work proposed a strategy based on mutual information maximization for Bayesian OSP, bypassing the necessity for a detailed, and often infeasible, combinatorial search.
Civera et al. [155]	2021	Multi-objective optimization	This paper proposed a novel approach using GAs and multi-objective optimization (MO) for a damage scenario-driven OSP method.
Sajedi and Liang [156]	2022	DGBO	This paper proposed a solution based on deep generative Bayesian optimization (DGBO) for parallel optimization of black-box/expensive error functions for OSP in SHM.
Mendler et al. [157]	2022	Fisher information	This paper presented a method for sensor placement using the Fisher information matrix for optimized sensor design, based on maximum damage detectability in the chosen structural elements.

**Table 12:** Advantages and disadvantages of local and global optimization algorithms.

Type	Refs.	Advantages	Disadvantages
Local	[173,174]	- Exact localization of optimal solutions. - High convergence speed. - High efficiency	- No escape from sub-optimal regions of the search space (the starting solution determines the optimization result).
Global	[175,176]	- Ability to escape from sub-optimal regions of the search space.	- Very low convergence speed, especially in the neighborhood of optimal solutions. - High optimization effort. - Uncertain quality of the optimization results.

**Table 16:** Analysis of the qualitative differences between DE, GA, and PSO.

Item	DE	GA	PSO
Provide a ranking system for solutions	No	Yes	No
Effect of population size on solution time	Linear	Exponential	Linear
Effects of best solutions on the population	Less	Medium	Most
Premature convergence tendency	Low	Medium	High
Ease of implementation	Medium	Easy	Medium
Density (continuity) of search area	More	Less	More
Applications in a variety of fields	Medium	Most	Medium
Ability to find good solutions without using local search	More	Less	More
Convergence is improved by homogeneous subgrouping	No	Yes	Yes

**Table 17:** Papers on optimization algorithms used for all levels of SHM and OSP (between 2010–2023).

Optimization Algorithm	Objective	Damage Type	Monitored System	Year	Ref.
Topology optimization	Damage detection and localization	Stiffness reduction	Composite laminate plate	2010	[283]
BFGS quasi-newton optimization	Damage detection	Variations in the structural variants	Space truss	2010	[284]
Novel multi-objective optimization	Damage detection and identification	Variations in the structural variants	Simple truss	2010	[285]
Modified effective independence distribution vector algorithm	OSP	Crack	High mobility multipurpose wheeled vehicle	2011	[286]
Innovative optimization	OSP	Variations in the structural variants	High-rise building	2011	[287]
Modified evolutionary algorithm based on covariance matrix adaption	Damage detection, localization, and quantification	Crack	Bridge columns	2011	[288]
Information entropy-based algorithm	OSP	Stiffness reduction	Skyscraper	2011	[289]
Improved charged system search algorithm	Damage detection	Stiffness reduction	Truss structures	2012	[290]
Improved evolutionary algorithm	Damage localization and evaluation	Crack	Shear wall and four-fixed supported plate	2012	[291]
Hybrid PSO-Simplex algorithm	Damage identification	Delamination	Composite beam	2012	[292]
Improved swarm intelligence algorithm	Damage detection	Crack	Steel frame	2012	[293]
Big Bang-Big Crunch algorithm	Damage detection	Stiffness reduction	Unbraced frame	2013	[294]
Multi-layer GA	Damage diagnosis	Stiffness reduction	Complex steel truss bridge	2013	[295]
Improved multi-particle swarm co-evolution optimization algorithm	Damage detection	Crack	Seven-story steel frame	2014	[296]
Continuous ant colony optimization algorithm	Damage detection and quantification	Stiffness reduction	Beam type structure	2014	[297]
Novel global artificial fish swarm algorithm	Damage detection	Crack	Building model	2014	[298]
Chaotic artificial bee colony algorithm	Damage identification	Variations in structural variants	Plate	2015	[299]
Improved PSO-NM algorithm	Damage detection and localization	Stiffness reduction	Two-storey frame	2015	[300]
Improved harmony search algorithm	Damage detection	Stiffness reduction	Wind turbine supporting structures	2015	[301]
Artificial bee colony algorithm with hybrid search strategy	Damage detection	Truss and plate	Stiffness reduction	2016	[302]
Improved differential evolution algorithm	Damage detection	Delamination	Composite beam and plate structures	2016	[303]
Modified adaptive harmony search algorithm	Damage detection and localization	Stiffness reduction	Beam-like and complex structures	2016	[304]
Inverse dynamics optimization algorithm	Damage detection	Stiffness reduction	Bridge	2017	[305]
Improved PSO	Damage detection	Stiffness reduction	Beam, truss and plate	2018	[306]
L1-norm optimization algorithm	Damage localization	Stiffness reduction	Metal beam and composite wind turbine	2018	[307]
Enhanced thermal exchange optimization algorithm	Damage identification	Stiffness reduction	Various structures	2018	[308]
Enhanced bat optimization algorithm	Damage detection, localization, and quantification	Variations in structural variants	Large-scale space structures	2019	[309]
Imperialist competitive algorithm	Damage detection, localization, and quantification	Variations in structural variants	Cantilever beam, continuous beam and plane portal frame	2019	[310]
Cuckoo search algorithm	Damage detection	Stiffness reduction	Bridges and beam-like structures	2019	[311]
Hybrid ant lion optimizer with improved Nelder–Mead algorithm	Damage detection, localization, and quantification	Stiffness reduction	Various structures	2020	[312]
Improved artificial bee colony algorithm	Damage detection	Crack	Beam	2020	[313]
Hybrid unified PSO	Damage assessment	Changes in vibration responses	Beam, plane truss and space truss	2020	[314]
Chimp optimization algorithm	Damage detection	Variations in structural variants	Steel truss	2021	[315]
Water strider algorithm	Damage detection	Variations in structural variants	Bridge	2021	[316]
Arithmetic optimization algorithm	Damage detection, localization and quantification	Variations in structural variants	Composite plates	2021	[317]
Gold rush optimization algorithm	Damage detection	Truss structures	Stiffness reduction	2022	[318]
Hybrid Whale-Chimp optimization algorithm	Damage detection	Stiffness reduction	Two-story rigid frame model and simply supported beam	2022	[319]
Hybrid butterfly optimization algorithm	Damage prediction	Crack	Beam	2022	[320]
Artificial Gorilla troops optimization algorithm	Damage detection	Stiffness reduction	Girder bridge	2023	[321]
Hybrid butterfly optimization algorithm	Damage prediction	Crack	Beam	2022	[320]
Artificial Gorilla troops optimization algorithm	Damage detection	Stiffness reduction	Girder bridge	2023	[321]
Hybrid firefly and PSO algorithms	Damage detection	Variations in structural variants	Large-scale truss bridge	2023	[322]
Chaos game optimization algorithm	Damage identification	Variations in structural variants	Steel and aluminum structures	2023	[323]

**Table 18:** Applications of optimization algorithms in OSP methods.

Ref	Year	Objective Function	Optimization Algorithm
Zhang et al. [229]	2014	$F=\sqrt{\frac{1}{n(n-1)}\sum_{i=1}^{n}\sum_{j=1}^{n}MAC_{ij}^{2}}$	IPSO
Sun and Büyüköztürk [147]	2015	$f_{1}\left(\theta \right)=\underset{i\neq j}{max}{\left\{MAC_{ij}\left(\varphi \right)\right\}}_{i,j=1,2,...,p}$	Discrete ABC
		$f_{2}\left(\theta \right)=\underset{\left(i\neq j\right)}{\sum_{i=1,j=1}^{p}}{\left[MAC_{ij}\left(\theta \right)\right]}^{2}$	
Yi et al. [324]	2015	$F=\underset{i\neq j}{max}\frac{{\left(\Phi_{i}^{T}\Phi_{j}\right)}^{2}}{\left(\Phi_{i}^{T}\Phi_{i}\right)\left(\Phi_{j}^{T}\Phi_{j}\right)}$	Adaptive MA
Downey et al. [325]	2018	$F=\left(1-\alpha \right)\frac{MAE(P)}{\overset{´}{MAE}}+\alpha \frac{\beta (P)}{\overset{´}{\beta }}$	Adaptive GA
Zan et al. [326]	2018	$\begin{array}{c} R=\\ min\left(\left(\sum_{j=1}^{M}\sum_{i=1}^{M}t_{ij}y_{j}\right),-\left(\sum_{j=1}^{M}\sum_{i=1}^{M}s_{ij}y_{j}\right),\left(\sum_{j=1}^{M}\sum_{i=1}^{M}c_{ij}y_{j}\right)\right) \end{array}$	PSO
Jaya et al. [327]	2020	$G=n\sigma_{\epsilon }^{2}+{∥\Phi^{l}∥}_{2}^{2}-\frac{{\left({∥\Phi^{l}∥}_{2}^{2}+\sigma_{\varepsilon }^{2}m\right)}^{2}}{\sigma_{N}^{2}\left(m+tr\left(\Phi_{d}{\left(\Phi_{s}^{T}\Phi_{s}\right)}^{-1}\Phi_{d}^{T}\right)\right)+{∥\Phi^{l}∥}_{2}^{2}}$	GA
Ponti et al. [328]	2021	$f_{1}\left(s\right)=\frac{1}{\left|A\right|}\sum_{a\in A}\bar{t_{a}}$	MOEA
		$f_{2}\left(s\right)=\sqrt{\frac{1}{\left|A\right|}\sum_{a\in A}{\left(\bar{t_{a}}-f_{1}\left(s\right)\right)}^{2}}$	
Yang et al. [51]	2022	$E\left(d\right)=\left(\mu_{d}-\Psi_{design}^{*}\right)\Phi \left(\frac{\mu_{d}-\Psi_{design}^{*}}{\sigma_{d}}\right)+\sigma_{d}\phi \left(\frac{\mu_{d}-\Psi_{design}^{*}}{\sigma_{d}}\right)$	Bayesian optimization algorithm
Saheb et al. [329]	2022	$f(i)=1/M\sum_{j=1}^{M}\sqrt{{\left(x_{i}-x_{j}\right)}^{2}+{\left(y_{i}-y_{j}\right)}^{2}}$	ACO and PSO
Goetschi et al. [330]	2022	$P=Max(1-\sum_{i\in G}P_{G}^{i}\prod_{j\in N}1-P_{D}^{i,j})$	PSO and GA

**Table 19:** Applications of optimization algorithms in SHM methods. (Note: E: Experimental application, A: Analytical application, One-stage: One-stage damage detection method, Multi-stage: Multi-stage damage detection method, AT: Application type; OA: Optimization algorithm).

Ref.	Year	Stage	Structure	AT	Objective Function	OA	Metric
Hao and Xia [331]	2002	One-stage	Cantilever beam	A	$J={∥W\{\Delta V^{A}\left(\{\alpha \right\})-\Delta V^{E}\}∥}^{2}$	GA	Modal data
					= ${\left\{\Delta V^{A}\left(\{\alpha \right\})-\Delta V^{E}\right\}}^{T}W^{2}\left\{\Delta V^{A}\left(\{\alpha \right\})-\Delta V^{E}\right\}$		
Braun et al. [332]	2015	One-stage	Spring-mass system	E and A	$\begin{array}{c} \epsilon =\\ \sum_{j=1}^{N}\left(\sum_{j=1}^{N_{t}}{\left[x_{j}^{Mod}\left(K,t_{i}\right)-x_{j}^{Exp}\left(K,t_{i}\right)\right]}^{2}\right) \end{array}$	ACO	Stiffness
Cha and Buyukozturk [58]	2015	Multi-stage	3D steel structures	A	$E=\sum_{i=1}^{ms}\left(\sum_{i=1}^{el}\left|\Phi_{i}^{dT}K_{j}\Phi_{i}^{d}-\Phi_{i}^{sT}K_{j}\Phi_{i}^{s}\right|\right)$	GA	Mode shape and stiffness
Vo-Duy et al. [333]	2016	Multi-stage	Composite plate	A	$\varepsilon \left(x\right)=\sum_{i=1}^{nm}\frac{∥\phi_{i}^{d}-\phi_{i}^{d}\left(x\right)∥}{∥\phi_{i}^{d}∥}$	DE	Mode shape
Gomes et al. [334]	2016	Multi-stage	Composite material	A and E	$\begin{array}{c} J=\sqrt{\frac{1}{N}\sum_{i=1}^{n}{\left(1-\frac{\omega_{i}^{real}}{\omega_{i}{\left(\vec{X}\right)}^{model}}\right)}^{2}}+\\ \sum_{i=1}^{n}{\left({\ddot{x}}^{\mathrm{real}\;}-{\ddot{x}}^{\mathrm{model}\;}\right)}^{2} \end{array}$	GA	Natural frequency and acceleration
Gui et al. [335]	2017	One-stage	Frame aluminum structure	E and A	$F{\left(x\right)}_{1}=\left(0.5{∥w∥}^{2}+C\sum_{i=1}^{N}\zeta_{i}\right)$	GA and PSO	Measured discrete signal
					$F{\left(x\right)}_{2}=sign\left(\sum_{i=1}^{N}\alpha_{i}y_{i}K\left(x,x_{i}\right)+b\right)$		
Gomes et al. [61]	2018	One-stage	CFRP plate	E and A	$J=\sqrt{\frac{1}{N}\sum_{i=1}^{n}{\left(1-\frac{\omega_{i}^{d}}{\omega_{i}{\left(\vec{x}\right)}^{c}}\right)}^{2}}$	GA	Natural frequency
Tran-Ngoc et al. [64]	2021	One-stage	Composite plate	A	$£\left(x\right)=\sqrt{\sum_{k=1}^{N_{k}}M_{k}^{2}-{\bar{M}}_{k}^{2}}$	HGACS	Natural frequency and mode shape
Ahmadi-Nedushan and Fathnejat [336]	2022	Multi-stage	Truss	A	$RMSD=\sqrt{\sum_{i=1}^{n}{\left[\left|\Delta P_{i}\right|-\left|\delta P{\left(X\right)}_{i}\right|\right]}^{2}}$	MTLBO	Modal strain energy
Hassani et al. [65]	2022	Multi-stage	Composite plate	A	$\mathrm{RMCE}:\;\underset{\left\{\alpha_{r}\right\},\;\left\{\beta_{r}\right\}}{min}\;∥\Psi -\Phi T∥$	PSO	Mode shape

## Data Availability

Not applicable.
